# Aberrant RON and MET Co-overexpression as Novel Prognostic Biomarkers of Shortened Patient Survival and Therapeutic Targets of Tyrosine Kinase Inhibitors in Pancreatic Cancer

**DOI:** 10.3389/fonc.2019.01377

**Published:** 2019-12-05

**Authors:** Chen-Yu Hu, Xiang-Ming Xu, Bo Hong, Zhi-Gang Wu, Yun Qian, Tian-Hao Weng, Yi-Zhi Liu, Tao-Ming Tang, Ming-Hai Wang, Hang-Ping Yao

**Affiliations:** ^1^State Key Laboratory for Diagnosis & Treatment of Infectious Diseases, The First Affiliated Hospital, Zhejiang University School of Medicine, Hangzhou, China; ^2^National Clinical Center for Diagnosis and Treatment of Infectious Disease, The First Affiliated Hospital, Zhejiang University School of Medicine, Hangzhou, China; ^3^Department of Colorectal Surgery, The First Affiliated Hospital, Zhejiang University School of Medicine, Hangzhou, China; ^4^Department of Pathology, The Second Affiliated Hospital, Hangzhou, China; ^5^Department of Clinical Laboratory, The Second Affiliated Hospital, Zhejiang University School of Medicine, Hangzhou, China; ^6^Cancer Biology Research Center, Amarillo, TX, United States; ^7^Department of Pharmaceutical Sciences, Texas Tech University Health Sciences Center School of Pharmacy, Amarillo, TX, United States

**Keywords:** pancreatic cancer, tyrosine kinase inhibitors, RON receptor tyrosine kinase, MET receptor tyrosine kinase, prognosis biomarker, therapeutic target

## Abstract

RON (recepteur d'origine nantais) and MET (hepatocyte growth factor receptor) are tyrosine kinase receptors. Various cancers have aberrant RON and MET expression and activation, which contribute to cancer cell proliferation, invasiveness, and metastasis. Here, we explored RON and MET expression in pancreatic cancer and their relationship with overall survival (OS) time, and evaluated their significance as therapeutic targets of tyrosine kinase inhibitors in pancreatic cancer. We enrolled 227 patients with pancreatic cancer in the study. RON and MET expression was analyzed by immunohistochemical staining. Four human pancreatic cancer cell lines expressing variable levels of RON or MET and four MET superfamily inhibitors (BMS777607, PHA665752, INCB28060, Tivantinib) were used. The effect of the four tyrosine kinase inhibitors on cell viability, migration, and apoptosis were determined using cell viability, scratch wound healing, and Caspase-Glo 3/7 assays. Cellular signaling was analyzed by immunoprecipitation and western blotting. The therapeutic efficacy of the tyrosine kinase inhibitors was determined with mouse xenograft pancreatic cancer models *in vivo*. There was wide aberrant RON and MET expression in the cancer tissues. In 227 pancreatic cancer samples, 33% had RON overexpression, 41% had MET overexpression, and 15.4% had RON and MET co-overexpression. RON and MET expression were highly correlated. RON and MET expression levels were significantly related to OS. Patients with RON and MET co-overexpression had poorer OS. BMS777607 and PHA665752 inhibited pancreatic cancer cell viability and migration, and promoted apoptosis by inhibiting RON and MET phosphorylation and further inhibiting the downstream signaling pathways *in vitro*. They also inhibited tumor growth and further inhibited phosphorylated (phosphor)-RON and phospho-MET expression in the mouse xenograft models *in vivo* effectively. INCB28060, which inhibits the MET signaling pathway alone, was not effective. RON and MET can be important indicators of prognosis in pancreatic cancer. Tyrosine kinase inhibitors targeting RON and MET in pancreatic cancer are a novel and potential approach for pancreatic cancer therapy.

## Introduction

Cancer currently poses a serious threat to human health. Advances in precision medicine have allowed many common cancers to be detected early and treated effectively, reducing mortality. As a leading cancer, pancreatic cancer has very high mortality. The 1-year survival rate of pancreatic cancer is <20%. Due to the lack of early diagnosis and effective treatment, the death rate from pancreatic cancer will become the second leading cause of cancer-related death in the next 20 years (1–3). Therefore, there is an urgent need to explore more therapeutic targets.

RON (macrophage-stimulating 1 receptor, MST1R) is a member of the MET proto-oncogene family, which also includes another member, MET (MET proto-oncogene, receptor tyrosine kinase) (4, 5). MET and RON were discovered in the early 1980s and early 1990s, respectively (4, 6, 7). The receptor tyrosine kinase (RTK) RON (7) and MET [also known as hepatocyte growth factor receptor [HGFR]; scatter factor (8)] are first produced as a single-chain ~180-kDa precursor, and then proteolytically cleaved to form a mature protein with two subunits: a ~40-kDa extracellular α-subunit with the Sema domain responsible for ligand binding, receptor dimerization, and phosphorylation; and a ~150-kDa transmembrane β-subunit with intrinsic tyrosine kinase activity, linked by a disulfide bond (7, 9). MSP [macrophage-stimulating protein, also known as MST1 and hepatocyte growth factor-like [HGFL]] is a RON ligand, and HGF is a MET ligand. Many studies on mouse cell models have shown that RON and MET play an important role in normal embryonic development and organogenesis, but their functions are restricted in adults (10–12). There is aberrant RON and MET expression and activation in various cancers, including gastric, prostate, ovarian, and breast cancer, and such aberrations contribute to cancer cell proliferation, invasiveness and metastasis (7, 13–15). Moreover, it has been experimentally confirmed that ligand-induced RON activation can transphosphorylate MET, and vice versa. Although the RON and MET ligands are different, they cross-talk and act synergistically in intracellular signaling (9, 16). The role of RON and MET in pancreatic cancer malignant progression, angiogenesis, and chemoresistance has also been studied extensively via genetic, biochemical, and biological models (17–21). In the present study, we mainly focused on the co-expression characteristics and pathological significance of RON and MET in pancreatic cancer tissues. At the same time, many tyrosine kinase inhibitors (TKIs) and targeted drugs that inhibit RON or MET are also being developed (22–25). However, few studies have compared the inhibitory effects of different TKIs targeting RON or MET in pancreatic cancer. Therefore, whether RON and MET can be used as important prognostic indicators and new therapeutic targets in pancreatic cancer is worth further exploration.

Here, we used a panel of pancreatic cancer cell lines expressing different levels of RON and MET as the model. The cell lines have previously been used to determine the role of RON and MET in regulating pancreatic cancer tumorigenic activity (22). Here, we used four MET superfamily TKIs to explore their therapeutic effects on pancreatic cancer: BMS777607, PHA665752, INCB28060 (capmatinib; INC280), and Tivantinib (ARQ 197). BMS777607 is a selective adenosine triphosphate (ATP)-competitive TKI with high specificity for RON at a median inhibitory concentration (IC50) of 1.8 nM, and targets MET with an IC50 of 3.9 nM (26). PHA665752 is a potent, selective, and ATP-competitive TKI of RON (IC50 68 nM) and MET (IC50 9 nM) (27). INCB28060 is a potent and selective TKI with high specificity for MET (IC50 0.13 nM) (28). Tivantinib (ARQ 197), a novel and highly selective non–ATP-competitive MET inhibitor, can inhibit human recombinant MET with a calculated inhibitory constant (Ki) of ~355 nM, and is currently in phase III cancer clinical trials (29, 30). Tivantinib (ARQ 197) exhibits anti-tumor activity independent of MET inhibition (31). Tivantinib can affect microtubule dynamics, induce G2/M arrest, and promote apoptosis (32, 33). As Tivantinib is the first MET-selective inhibitor to be used in advanced human clinical trials, we used it as a control inhibitor in the present study.

We explored RON and MET expression in pancreatic cancer and their relationship with survival time to investigate whether they can be used as a new prognostic marker in pancreatic cancer. Then, we explored the signaling pathways of aberrant RON and MET expression in pancreatic cancer, and evaluated the significance of RON and MET as therapeutic targets of TKIs targeting RON and/or MET in pancreatic cancer, further providing new means and therapy for treating pancreatic cancer in the future.

## Materials and Methods

### Cell Lines and Reagents

Human pancreatic cancer cell lines expressing variable levels of RON and MET were selected as the TKI targeting model. The BxPC3 [KRAS proto-oncogene, GTPase wild-type [KRAS wt]], AsPC1 [KRAS [mutant, mut]], and Panc1 [KRAS [mut]] cell lines were from American Type Cell Culture (ATCC, Manassas, VA, USA) and had been authenticated in 2010 with cytogenesis. The L3.6p1 cell line was provided by G.E. Gallick (University of Texas MD Anderson Cancer Center, Houston, TX, USA) (34). Flow cytometric and western blot analysis showed that the BxPC3 and AsPC1 cells had RON and MET co-expression. L3.6p1 cells express RON alone and Panc1 cells express MET alone (Figure S1). The cell lines were cultured in their appropriate culture medium supplemented with 10% fetal bovine serum. Anti-RON monoclonal antibodies (mAbs, Zt/g4, Zt/f2) and rabbit polyclonal immunoglobulin G (IgG) antibody R5029, specific to the RON C-terminus, were used as previously described (23, 25, 35, 36). Phosphorylated (phospho)-tyrosine mouse mAb (P-Tyr-100, Cat# 9411) and rabbit antibodies to extracellular signal–regulated kinase 1/2 (ERK1/2) (Cat# 4695), AKT (Cat# 4685), phospho-ERK1/2 (p44/42) (Cat# 4376), and phospho-AKT (Cat# 4060) were from Cell Signaling Technology (Beverly, MA, USA). MET-specific rabbit IgG antibodies (Cat# ab51067) were from Abcam (Burlingame, CA, USA). BMS777607, PHA665752, INCB28060, and Tivantinib were from MedChem Express (Monmouth Junction, NJ, USA) and stored at a concentration of 10 mM in dimethyl sulfoxide (DMSO). Recombinant human MSP (Cat# 352-MS), recombinant human HGF (Cat# 294-HG), and the DuoSet IC human phospho-RON ELISA (enzyme-linked immunosorbent assay, cat. no. DYC1947-5) kit were from R&D Systems (Minneapolis, MN, USA).

### Patients and Tissue Specimens

We analyzed 227 patients who had been pathologically diagnosed with pancreatic cancer with or without liver metastases between January 2010 and June 2015 at the Affiliated Hospital, Zhejiang University School of Medicine. All patients underwent pathological biopsy for pancreatic cancer surgery. The clinical parameters included patient demographics, tumor-node-metastasis (TNM) stage, tumor differentiation, tumor size, and treatment modality. Furthermore, 20 patients with benign pancreatic or liver disease, such as pancreatitis and cysts, and hepatolithiasis, were enrolled as a control group. All tissues were fixed in 10% buffered formalin and embedded in paraffin. This study was approved by the Ethics Committee of the First Affiliated Hospital (reference numbers: 2017427-1), Zhejiang University School of Medicine.

### IHC Staining and Evaluation of RON and MET in Pancreatic Cancer Tissue

In human pancreatic cancer tissue, IHC staining was carried out using Zt/f2 (5 μg/mL) as the primary antibody for RON and rabbit anti-MET mAb (1:100, 51,067, abcam) for MET, followed by EnVision System reagents (Dako, Carpentaria, CA, USA) as previously described (35). The negative control was performed by replacing the primary antibody with isotype-matched mouse IgG (5 μg/mL) (Figure S3). Human normal/benign pancreas tissue were used as the negative control. Two pathologists without knowledge of the patients' clinical records examined and scored the sections. Five tumor fields under ×400 magnification were randomly selected. Cytoplasmic and/or tumor cell membrane staining were considered to indicate positive expression. RON and MET expression were determined using a semiquantitative system as previously described (35). The proportion of positive cells was scored as follows: 0 (<5%), 1 (6–25%), 2 (26–50%), 3 (51–75%), and 4 (>75%). The staining level was evaluated as follows: 0 (no staining), 1 (weak staining, light yellow), 2 (moderate staining, yellowish brown), and 3 (strong staining, brown). The sum score, determined by adding up the positive proportion score and the staining level score, was as follows: 0 (negative; 0+), 1–3 (weakly positive; 1+), 4–5 (moderately positive; 2+), and 6–7 (strongly positive; 3+).

### Cell Viability and Caspase-Glo 3/7 Assays

Pancreatic cancer cells (1 × 10^4^ cells per well in a 96-well plate in triplicate) were incubated in a 5% CO_2_ atmosphere at 37°C. Cell viability at 24, 48, and 72 h after TKI treatment (0–15 μM) was determined using Cell Counting Kit-8 (CCK-8, 10 μL/well, cat. no. HY-K0301-100T; MedChem Express). BxPC3 cell apoptosis following TKI treatment (0–10 μM) was measured using an ApoLive-Glo Multiplex Assay kit (size: 5 × 10 mL, cat. no. #G6411; Promega, Madison, WI, USA) to detect caspase-3/7 activity in the cells according to the manufacturer's instructions.

### Cell Migration Assays

The effect of the TKIs on pancreatic cancer cell migration was detected using a wound healing assay. Pancreatic cancer cells (5 × 10^5^ cells) were seeded in 6-well plates and allowed to grow until 100% confluent. A scratch was made in the plate using a P200 pipette tip after the inhibitors (1.5 μM BMS777607, 5 μM INCB28060, 1.5 μM PHA665752, 0.15 μM Tivantinib) had been added. According to the CCK-8 experimental results at 24 h (Figure S2), the cell viability rate under the above drug concentration was >80%. Images were collected at 0 and 24 h under an inverted microscope (Ziess, Oberkochen, Germany). Cell migration was analyzed using National Institutes of Health (NIH, Bethesda, MD, USA) ImageJ software and GraphPad 7 (GraphPad Software, San Diego, USA).

### Phosphorylation, Immunoprecipitation, and Western Blotting

These steps were conducted as previously described (23, 25). The phosphorylation assay was performed by stimulating BxPC3 cells (2 × 10^6^ cells/mL/sample) with 2 nM MSP (RON activation) and 2 nM HGF (MET activation), followed by TKIs (5 μM BMS777607, 5 μM INCB28060, 5 μM PHA665752, 0.5 μM Tivantinib) at 37°C for 60 min (25). Cellular proteins from cell lysates (30 μg per sample) and tissue lysates (50 μg per sample) were separated in 8% sodium dodecyl sulfate–polyacrylamide gel electrophoresis (SDS-PAGE) under reduced conditions. RON, MET, or other signaling proteins were detected by western blotting using R5029, ab51067, or the corresponding antibodies, visualized using enhanced chemiluminescence reagents and were analyzed using the VersaDoc MP 5000 Imaging system (Bio-Rad). The membranes also were reprobed with antibodies to GAPDH (glyceraldehyde-3-phosphate dehydrogenase) to ensure equal sample loading.

For immunoprecipitation, cellular proteins (250 μg per sample) were mixed with anti-phospho-tyrosine PY-100 (1:100) coupled to protein G Sepharose beads. Proteins were separated in 8% SDS-PAGE under reduced conditions. Phospho-RON or phospho-MET was detected by western blotting using R5029 or ab51067.

### Human Phospho-RON ELISA

The DuoSet IC ELISA was used for measuring human phospho-RON in cell lysates according to the manufacturer's instructions. Capture antibody (mouse anti-human RON, 8.0 μg/mL in phosphate-buffered saline [PBS]) was coated in a 96-well microplate and incubated overnight at room temperature. Then, cell lysates (30 μg/100 μL/well) were added and incubated for 2 h at room temperature, followed by the addition of diluted anti-phospho-tyrosine PY-100–horseradish peroxidase (HRP) to each well. After adding substrate solution, the absorbance of each well was measured using a microplate reader (Bio-Tek ELx800) at 450 nm wavelength. The phospho-RON levels in the cell lysates were evaluated based on the optical density value.

### Pancreatic Cancer Xenograft Model and TKI Treatment

All mouse experiments were approved by the institutional animal care committee (reference numbers: 2017400-1). Female athymic nude mice (6 weeks old, Taconic, Cranbury, NJ) were acclimated to the animal housing facility for at least 1 week before the study. Then, the mice were injected with 5 × 10^6^ BxPC3, AsPC1, L3.6p1, or Panc1 cells in the subcutaneous space of the right flank as previously described (37, 38). The mice were randomized to different groups (*n* = 4 per group). Treatment began when all tumors had a mean volume of ~100 mm^3^. BMS777607, INCB28060, PHA665752, or Tivantinib was administered by gavage at 25, 5, 25, and 20 mg/kg daily per mouse, respectively, and continued for 14 days. Control mice were injected with vehicle (DMSO in PBS). Tumor volume and mouse body weights were recorded every 4 days. The volume (V) of the subcutaneous tumors was calculated as follows: V = (length × width^2^)/2. The animals were euthanized if the tumors became necrotic or ulcerated through the skin or when tumor volumes were >2,000 mm^3^ or if the mice bred for >60 days after becoming tumor-burdened. The tumors were harvested for the subsequent experiments.

### Data Analysis and Statistical Significance

Statistical analysis was performed using SPSS (v17.0; IBM Corporation, Armonk, NY, USA) and GraphPad 7. The relationship between RON and MET expression and clinicopathological characteristics was compared using the chi-square test. Overall survival (OS) was calculated from the diagnosis of pancreatic cancer until death or the date of the last follow-up. Survival data were analyzed by the Kaplan–Meier method and log rank test. The independent prognostic factors of survival were identified using Cox proportional hazard model analysis. The significance of the experimental and control groups was analyzed using one-way analysis of variance (ANOVA) or the two independent samples *t*-test. Results are shown as the mean ± SD. *P* < 0.05 was considered statistically significant.

## Results

### RON and MET Expression in Pancreatic Cancer and Their Relationship With Clinicopathological Characteristics

A total of 227 patients (156 men and 71 women) with pancreatic cancer were enrolled in the study. All patients were followed up until December 2018, when only 10 patients were confirmed to be still alive. The median age at tumorectomy was 63 years (range, 26–93 years). All patients were diagnosed with infiltrating ductal adenocarcinoma. Table 1 summarizes the characteristics of the patient population.

**Table 1:** Correlation between RON/MET expression and clinical characteristics of patients with pancreatic cancer.

**Characteristic**	**Cases**	**RON expression (*****P*****)**			***P*-value**	**MET expression (*****P*****)**			***P*-value**
		**Overexpressed**	**Weak/moderate**	**Negative**		**Overexpressed**	**Weak/moderate**	**Negative**	
**Age (years)**		(χ^2^ = 0.562, *P* >0.05)			0.755	*(χ*^2^ = 1.562, *P* > 0.05)			0.458
<63	111 (48.9%)	38 (34.2%)	56 (50.5%)	17 (15.3%)		42 (37.8%)	57 (51.4%)	12 (10.8%)	
≥63	116 (51.1%)	37 (31.9%)	64 (55.2%)	15 (12.9%)		51 (44.0%)	57 (49.1%)	8 (6.9%)	
**Sex**		*(χ*^2^ = 1.718, *P* > 0.05)			0.424	(χ^2^ = 0.074, *P* > 0.05)			0.963
Male	156 (68.7%)	48 (30.8%)	87 (55.8%)	21 (13.5%)		63 (40.4%)	79 (50.6%)	14 (9.0%)	
Female	71 (31.3%)	27 (30.8%)	33 (46.5%)	11 (15.5%)		30 (42.3%)	35 (49.3%)	6 (8.5%)	
**Tumor size**		(χ^2^ = 2.617, *P* > 0.05)			0.270	(χ^2^ = 7.304, *P* < 0.05)			**0.026**
1 ~ 2	71 (31.3%)	19 (26.8%)	39 (54.9%)	13 (18.3%)		21 (29.6%)	40 (56.3%)	10 (14.1%)	
3 ~ 4	156 (68.7%)	56 (35.9%)	81 (51.9%)	19 (12.2%)		72 (46.2%)	74 (47.4%)	10 (6.4%)	
**Lymph node metastasis**		(χ^2^ = 1.730, *P* > 0.05)			0.421	(χ^2^ = 0.005, *P* > 0.05)			0.997
Yes	103 (45.4%)	30 (29.1%)	56 (54.4%)	17 (16.5%)		42 (40.8%)	52 (50.5%)	9 (8.7%)	
None	124 (54.6%)	45 (36.3%)	64 (51.6%)	15 (12.1%)		51 (41.1%)	62 (50.0%)	11 (8.9%)	
**Distant metastasis**		(χ^2^ = 7.938, *P* < 0.05)			**0.019**	(χ^2^ = 4.873, *P* > 0.05)			0.087
Yes	59 (26.0%)	28 (47.5%)	23 (39.0%)	8 (13.6%)		30 (50.8%)	27 (45.8%)	2 (3.4%)	
None	168 (74.0%)	47 (28.0%)	97 (57.7%)	24 (14.3%)		63 (37.5%)	87 (51.8%)	18 (10.7%)	
**TNM stage**		(χ^2^ = 3.051, *P* > 0.05)			0.217	(χ^2^ = 4.163, *P* > 0.05)			0.125
1 ~ 2	150 (66.0%)	44 (29.3%)	85 (56.7%)	21 (14.0%)		57 (38.0%)	76 (50.7%)	17 (11.3%)	
3 ~ 4	77 (34.0%)	31 (40.3%)	35 (45.5%)	11 (14.3%)		36 (46.8%)	38 (49.4%)	3 (3.9%)	
**Differentiation**		(χ^2^ = 7.983, *P* > 0.05)			0.092	(χ^2^ = 1.341, *P* > 0.05)			0.854
Well	25 (11.0%)	8 (32.0%)	15 (60.0%)	2 (8.0%)		11 (44.0%)	13 (52.0%)	1 (4.0%)	
Moderate	170 (74.9%)	50 (29.4%)	94 (55.3%)	26 (15.3%)		68 (40.0%)	85 (50.0%)	17 (10.0%)	
Poor	32 (14.1%)	17 (53.1%)	11 (34.4%)	4 (12.5%)		14 (43.8%)	16 (50.0%)	2 (6.3%)	
**Treatment**		(χ^2^ = 4.886, *P* > 0.05)			0.087	(χ^2^ = 2.836, *P* > 0.05)			0.242
Chemotherapy	95 (41.9%)	31 (32.6%)	56 (58.9%)	8 (8.4%)		45 (47.4%)	42 (44.2%)	8 (8.4%)	
None	132 (58.1%)	44 (33.3%)	64 (48.5%)	24 (18.2%)		48 (36.4%)	72 (54.5%)	12 (9.1%)	
**MET expression**		(χ^2^ = 14.303, *P* < 0.05)			**0.006**				
Overexpressed	93 (41.0%)	35 (37.6%)	53 (57.0%)	5 (5.4%)					
Weak/moderate	114 (50.2%)	35 (30.7%)	59 (51.8%)	20 (17.5%)					
Negative	20 (8.8%)	5 (25.0%)	8 (40.0%)	7 (35.0%)					

*Bold values means statistically significant*.

**Figure 1:**
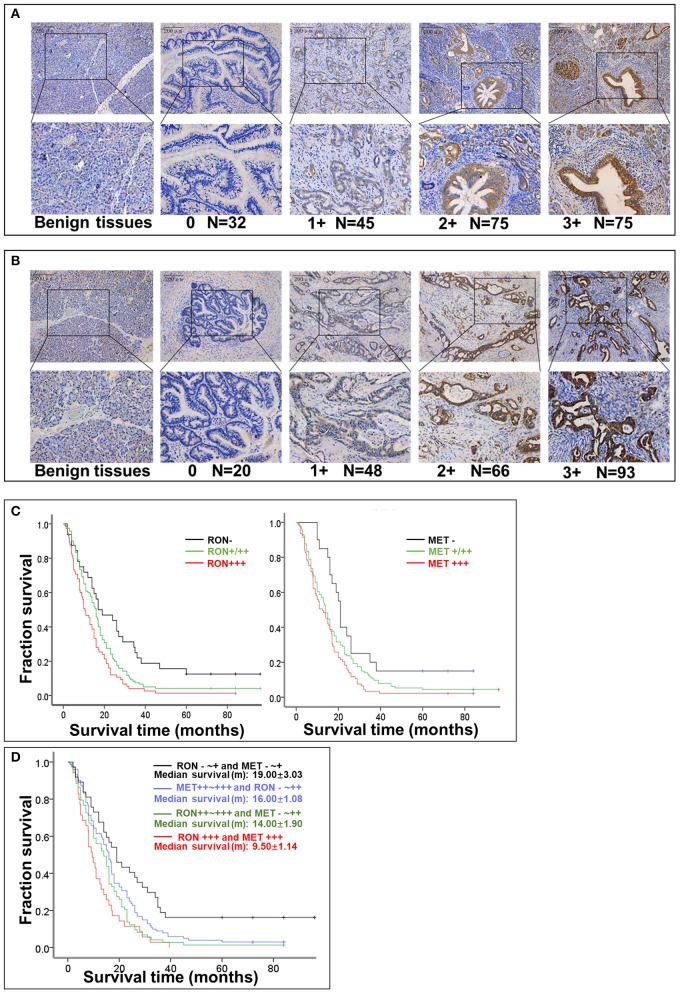
Relationship between RON and MET expression levels in pancreatic cancer tissue samples and OS. **(A)** RON expression level in benign tissues and pancreatic cancer tissues. Original magnification ×100 (all photomicrographs). **(B)** MET expression level in benign tissues and pancreatic cancer tissue. **(C)** Kaplan–Meier plots with log-rank test analysis of the relationship between RON or MET expression level and survival. **(D)** Relationship between RON and MET co-expression and survival.

RON and MET staining was detected in the cell membrane and cytoplasm in cancerous and non-cancerous cells in patients with pancreatic cancer (Figures 1A,B). The pancreatic cancer samples had significantly higher proportions of positive RON expression (195 of 227 patients, 85.9%) and MET expression (207 of 227 patients, 91.2%) compared with the normal/benign pancreatic tissue samples (5 of 20 patients, 25%). Moreover, the positive samples in the normal/benign pancreatic tissues all had weak expression. Of the 227 pancreatic cancer samples, 33% had RON overexpression (3+), 41% had MET overexpression (3+), and 15.4% had RON and MET co-overexpression. Compared to RON, MET was more widely overexpressed in pancreatic cancer tissues. Table 1 shows the relationship between RON and MET expression and the clinicopathological characteristics. Elevated RON and MET expression was associated with distant metastasis (*p* < 0.05) and tumor size (*p* < 0.05), respectively. RON expression level was highly correlated with MET expression (*p* < 0.01). However, RON and MET expression was not associated with patient age at tumorectomy (<65 vs. ≥65 years), gender, lymph node metastasis, tumor-node-metastasis (TNM) stage, or chemotherapy treatment.

### Relationship Between RON and MET Expression and OS

The role of RON and MET expression in OS was assessed using Kaplan–Meier analysis and the log-rank test (Table 2). At the final follow-up, the median OS of the 227 samples was 15.0 ± 0.77 months [95% confidence interval [CI], 13.48–16.52 months]. RON and MET expression levels were significantly related to OS (*p* < 0.01). Patients with RON overexpression had poorer OS compared with patients with weak/moderate or negative RON expression (*p* < 0.01) (Figure 1C). The correlation between MET expression and OS was similar to that of RON (*p* < 0.01) (Figure 1C). Patients with RON and MET co-overexpression (median survival: 9.50 ± 1.14 months) had significantly shorter survival than patients with RON or MET weak and negative expression (median survival: 19.00 ± 3.03 months) (*p* < 0.01) (Figure 1D). As novel tumor biomarkers, RON and MET as combined indicators of prognosis for predicting patient survival may have more clinical evaluation value than RON or MET alone. The other prognostic factors associated with decreased OS were age (*p* < 0.01), tumor size (*p* < 0.01), lymph node metastasis (*p* < 0.05), distant metastasis (*p* < 0.01), TNM stage (*p* < 0.01), and chemotherapy treatment (*p* < 0.05).

**Table 2:** Univariate analysis of overall survival.

**Characteristic**	**Cases**	**Median survival (months)**	**95% CI (months)**	***P*-value**
**Age (years)**				
<63	111	16.000 ± 1.109	13.827–18.173	0.002
≥63	116	11.000 ± 1.498	8.064–13.936	
**Sex**				
Male	156	15.500 ± 0.812	13.909–17.091	0.565
Female	71	12.500 ± 1.302	9.948–15.052	
**Tumor size**				
1 ~ 2	71	20.000 ± 1.149	17.748–22.252	0.000
3 ~ 4	156	10.000 ± 1.171	7.705–12.295	
**Lymph node metastasis**				
Yes	103	11.000 ± 1.267	8.516–13.484	0.010
None	124	16.000 ± 0.879	14.277–17.723	
**Distant metastasis**				
Yes	59	7.000 ± 0.524	5.972–8.028	0.000
None	168	17.000 ± 0.647	15.731–18.269	
**TNM stage**				
1 ~ 2	150	17.000 ± 0.612	15.801–18.199	0.000
3 ~ 4	77	7.900 ± 0.797	6.338–9.462	
**Differentiation**				
Well	25	16.500 ± 2.998	10.625–22.375	0.216
Moderate	170	15.000 ± 0.848	13.338–16.662	
Poor	32	9.900 ± 2.828	4.356–15.444	
**Treatment**				
Chemotherapy	95	17.100 ± 0.677	15.774–18.426	0.035
None	132	11.000 ± 1.092	8.859–13.141	
**RON expression**				
Overexpressed	75	10.600 ± 1.378	7.900–13.300	0.001
Weak/moderate	120	16.000 ± 1.128	13.789–18.211	
Negative	32	17.000 ± 5.657	5.913–28.087	
**MET expression**				
Overexpressed	93	12.500 ± 1.854	8.865–16.135	0.006
Weak/moderate	114	14.000 ± 1.143	11.759–16.241	
Negative	20	21.000 ± 0.730	19.569–22.431	

In multivariate analysis, patients with RON and MET co-expression had much higher relative risk (RR: 1.664; 95% CI, 1.169–2.369; *p* < 0.01) than patients with RON or MET negative expression. Patients with RON expression had much higher RR (RR: 1.911; 95% CI, 1.271–2.875; *p* < 0.01) than the RON-negative control. The same result was obtained for MET expression (RR: 1.967; 95% CI, 1.193–3.244; *p* < 0.01). In addition, distant metastasis (*p* < 0.01), age (*p* < 0.05), tumor size (*p* < 0.01), and chemotherapy (*p* < 0.01) remained independent prognostic factors of poor OS (Table S1).

### Effect of TKIs *in vitro* on Pancreatic Cancer Cell Viability, Migration, and Apoptosis

**Figure 2:**
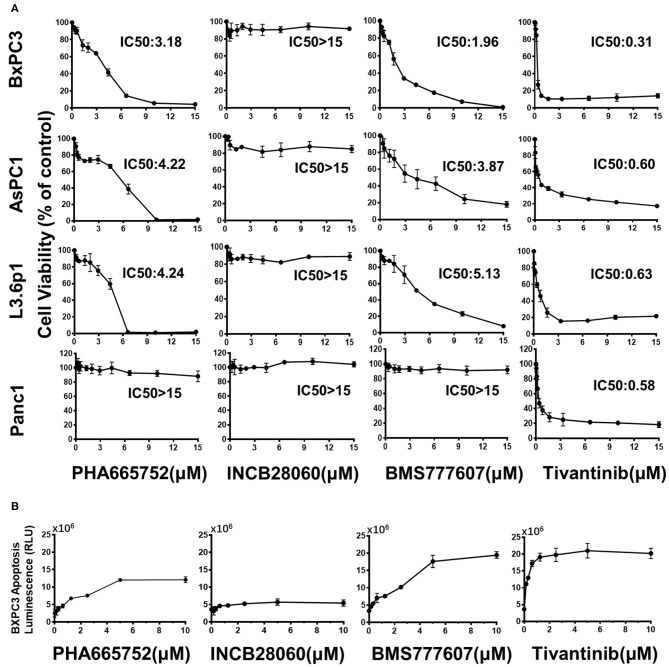
Effect of TKIs on pancreatic cancer cell viability and apoptosis. **(A)** Pancreatic cancer cells (1 × 10^4^ cells per well in a 96-well plate in triplicate) were incubated in a 5% CO_2_ atmosphere at 37°C. The cells were treated with TKIs (0–15 μM) for 72 h. CCK-8 measurement of TKI-treated pancreatic cancer cell viability. Control cell viability was defined as 100% and was used to calculate the percentages of cell viability in drug-treated cells. **(B)** Caspase-Glo 3/7 assay measurement of TKI-treated BxPC3 cell apoptosis. The cells were treated with TKIs (0–10 μM) for 72 h. Caspase-Glo 3/7 Reagent (25 μl) was added to each well and briefly mixed by orbital shaking (1,300–1,500 rpm for ~30 s), incubated for 30 min at room temperature, and the luminescence was measured. Data shown are from one of three experiments with similar results.

In BxPC3 cells, TKI treatment reduced cell viability significantly in a time- and dose-dependent manner, except for INCB28060 (Figure S2). There was >80% reduction in cell viability when BxPC3 cells were treated with 15 μM BMS777607, 15 μM PHA665752, or 15 μM Tivantinib at 72 h, and only <20% reduction in cell viability when the cells were treated with 15 μM INCB28060. The calculated IC50 values of BMS777607, PHA665752, and Tivantinib at 72 h were 1.96, 3.18, and 0.31 μM, respectively. Although the IC50 value in BxPC3 cell viability differed, increasing the concentration of BMS777607 and PHA665752 had the same inhibitory effect as Tivantinib (Figure 2A). The TKIs had the same effect on AsPC1 cell viability as that on BxPC3 cells, and the calculated IC50 values of BMS777607, PHA665752, and Tivantinib at 72 h were 3.87, 4.22, and 0.60 μM, respectively (Figure S2). In L3.6p1 cells, the calculated IC50 values of BMS777607, PHA665752, and Tivantinib at 72 h were 5.13, 4.24, and 0.63 μM, respectively. In Panc1 cells, only Tivantinib affected cell viability (IC50 of 0.58 μM) (Figure 2A).

The Caspase-Glo 3/7 assays showed that Tivantinib, BMS777607, and PHA665752 promoted BxPC3 cell apoptosis in dose-dependently. In contrast, INCB28060 had low effects on BxPC3 cell apoptosis (Figure 2B).

**Figure 3:**
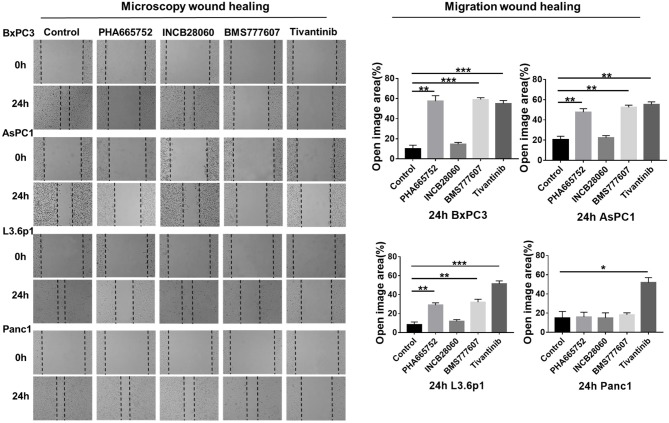
Effect of TKIs on pancreatic cancer cell migration *in vitro*. Pancreatic cancer cells (5 × 10^5^ cells) were seeded in 6-well plates and allowed to grow until 100% confluent. A scratch was made in the plate using a P200 pipette tip after the inhibitors (1.5 μM BMS-777607, 5 μM INCB28060, 1.5 μM PHA665752, 0.15 μM Tivantinib) had been added. Images were collected at 0 and 24 h, and cell migration was analyzed using ImageJ and GraphPad 7. Data shown are from one of three experiments with similar results. *p < 0.01, **p < 0.001, ***p < 0.0001.

Enhanced cell motility and invasiveness are important features of tumor progression. Therefore, we used the wound healing assay to investigate the effect of the TKIs on cell migration and invasion. After 24 h, wound healing was significantly reduced (*n* = 3, 2-tailed unpaired *t*-test, *p* < 0.001) in BxPC3 cells treated with 1.5 μM PHA665752, 1.5 μM BMS777607, and 0.15 μM tivantinib, with 57.49, 59.12, and 55.07% open area, respectively, compared to the scrambled control vector set with 10.07% open area. AsPC1 cells yielded similar results to that of the BxPC3 cells. Wound healing was also reduced (*p* < 0.001) in L3.6p1 cells treated with 1.5 μM PHA665752 and 1.5 μM BMS777607, with 28.98 and 32.07% open area, respectively, compared to the 8.26% of the empty vector. In Panc1 cells, only Tivantinib reduced cell migration significantly compared to scrambled control at 24 h (*p* < 0.01). Compared with the other three inhibitors, INCB28060 had no inhibitory effect on pancreatic cancer cell migration after 24 h (Figure 3).

From the above results, we concluded that BMS777607 and PHA665752, both targeting RON and MET, can significantly inhibit pancreatic cancer cell viability and migration and promote apoptosis. At the same time, INCB28060, targeting MET, had almost no effect on pancreatic cancer cell viability, migration, and apoptosis. Tivantinib exhibited a good inhibitory effect on pancreatic cancer cells independently of MET inhibition.

### Effect of TKIs on Cellular Signaling

**Figure 4:**
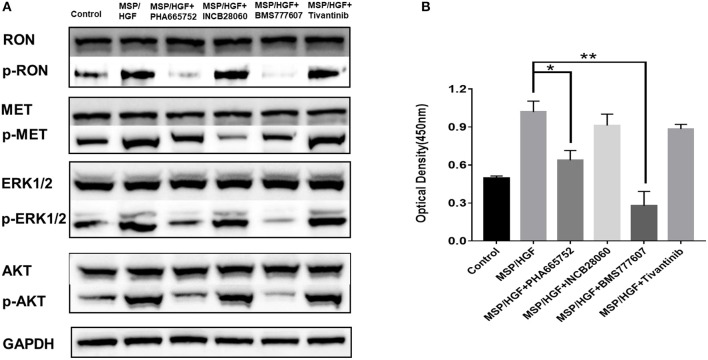
Effect of TKIs on cellular signaling. **(A)** Western blot of BxPC3 cells treated MSP, HGF, and TKIs (5 μM BMS777607, 5 μM INCB28060, 5 μM PHA665752, 0.5 μM Tivantinib). Proteins analyzed include RON, MET, AKT, ERK1/2, phospho-AKT, and phospho-ERK1/2. The membranes were also reprobed for GAPDH as the loading control. Phosphorylated RON and MET were analyzed by western blotting after immunoprecipitation. **(B)** ELISA measurement of phosphorylated human RON in BxPC3 cells as above. *p < 0.01, **p < 0.001.

The requirement of RON and MET for downstream signaling activation and cellular functions was studied using small-molecule TKIs in BxPC3 cells. BxPC3 cells were treated with 2 nM MSP and 2 nM HGF, followed by TKIs (5 μM BMS-777607, 5 μM INCB28060, 5 μM PHA665752, 0.5 μM Tivantinib) for 60 min at 37°C, and then phospho-RON and phospho-MET expression were analyzed by western blotting. MSP and HGF significantly stimulated RON and MET phosphorylation as compared to the control. BMS777607 and PHA665752 significantly silenced phospho-RON and phospho-MET expression. Moreover, inhibiting phospho-RON and phospho-MET expression significantly diminished both AKT and ERK1/2 phosphorylation, but had no effect on their protein expression. INCB28060 only inhibited phospho-MET expression, and had no effect on the downstream signaling pathway. Therefore, RON and MET signaling is critical in AKT and ERK1/2 activation, and BMS-777607 and PHA665752 might inhibit RON- and MET-mediated tumorigenesis (Figure 4A).

The same cell lysates treated with MSP, HGF, and inhibitors were also analyzed by ELISA to measure phospho-RON expression. The BMS777607 and PHA665752 group had significantly diminished optical density values (*P* < 0.01) compared to the MSP-alone group. These results corroborated BMS777607 and PHA665752 significantly decreasing phospho-RON and phospho-MET expression (Figure 4B).

### Therapeutic Effect of TKIs *in vivo* Inhibition of Xenograft Pancreatic Cancer Growth

Inoculating pancreatic cancer cells (5 × 10^6^ cells per mouse) into the mammary fat pad caused tumor growth in a time-dependent manner. The pancreatic cancer cells were oncogenic in the nude mice and rapidly caused tumor formation and growth *in vivo*. Treatment of the mice bearing pancreatic cancer xenografts (~100 mm^3^) was initiated with repeated injection of 20 mg/kg/day Tivantinib, 5 mg/kg/day INCB28060, 25 mg/kg/day BMS777607, or 25 mg/kg/day PHA665752, with a total of 14 doses for 14 days. In BxPC3 xenografts, Tivantinib, PHA665752, and BMS777607 inhibited BxPC3 cell–mediated tumor growth in a time-dependent manner. Significantly reduced (*p* < 0.0001) tumor volume was observed after Tivantinib, BMS777607 and PHA665752 treatment at day 20. At day 27, the mice in the control and INCB28060 groups were euthanized when tumor volumes exceeded 2,000 mm^3^. Compared to the control group, the PHA665752-, BMS777607-, and Tivantinib-treated mice had 52.32, 70.36, and 64.0% reduced tumor growth, respectively (**Figure 6A**). Then, at day 37, 43, and 47, mice in the PHA665752, Tivantinib, and BMS777607 groups were euthanized when tumor volumes exceeded 2,000 mm^3^. Compared with the control, BMS777607, Tivantinib, and PHA665752 extended the life of the mice by 20, 16, and 10 days, respectively (**Figure 6A**).

**Figure 5:**
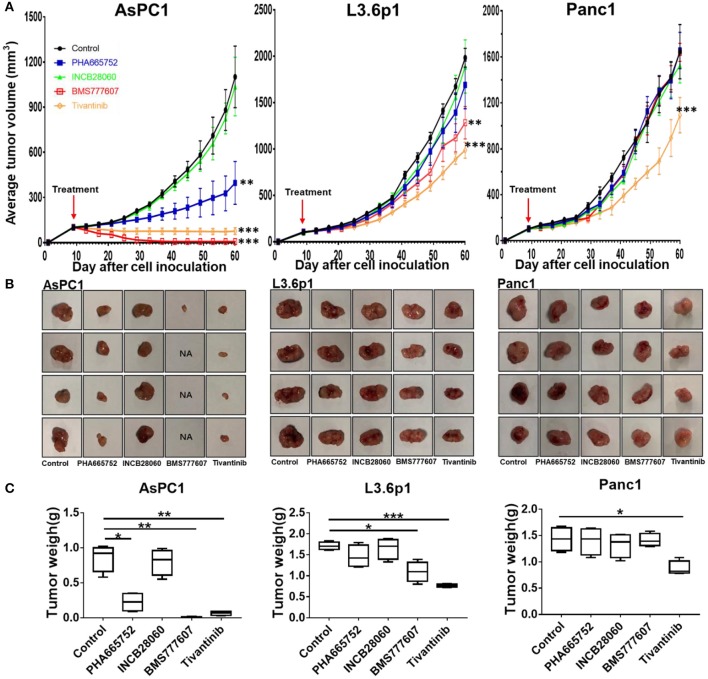
Therapeutic efficacy of TKIs in pancreatic cancer xenograft tumor models *in vivo*. **(A)** Average tumor volumes from nude mice (*n* = 4 per group) inoculated with 5 × 10^6^ AsPC1, L3.6p1, or Panc1 cells. **(B)** Individual tumors from each treatment group. NA, no tumors were observed in the injected site. **(C)** Tumor weights of nude mice (*n* = 4 per group) inoculated with AsPC1, L3.6p1, or Panc1 cells. *p < 0.01, **p < 0.001, ***p < 0.0001.

Due to the slower tumor growth speed of the AsPC1 xenograft tumor model as compared to the BxPC3 xenograft tumor model, we started the treatment at day 9. At day 60, all mice were euthanized. The tumor size and mouse body weight of the control and INCB28060 groups were not significantly different (Figure 5). The tumor weights of the PHA665752-treated mice (0.22 ± 0.14 g) and BMS777607-treated mice (0.06 ± 0.03 g) were much lower than that of the control group (0.86 ± 0.19 g, *p* < 0.01) (Figure 5C). Compared to the control group, the PHA665752- and BMS777607-treated mice had 74.42 and 93.02% tumor weight reduction, respectively. There were hardly any tumors in the BMS777607 group (Figure 5B). And the tumor volume of the PHA665752- and BMS777607-treated mice were much lower than that of the control group.

In L3.6p1 xenografts, BMS777607, and Tivantinib had a significant inhibitory effect on tumor growth, and average inhibition in tumor weight with statistical difference (*p* < 0.01) was 35.67 and 54.97% in the BMS777607 and Tivantinib groups, respectively (Figures 5A,C). In Panc1 xenografts, only Tivantinib had an obvious inhibitory effect as compared to the control, and the Tivantinib group had much lower average tumor weight (0.88 ± 0.13 g) than the control group (1.43 ± 0.22 g, *p* < 0.01) (Figure 5C). During the TKI treatment, all three groups of mice remained healthy and gained weight.

**Figure 6:**
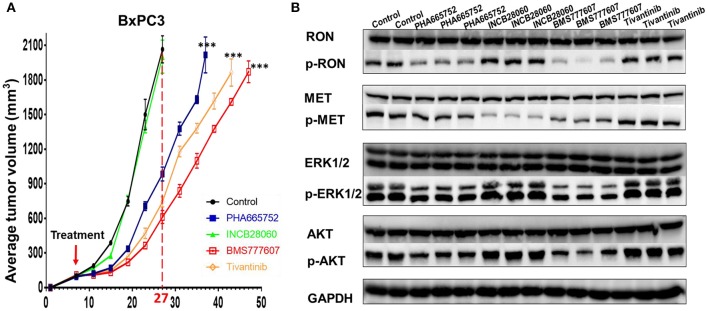
Effect of TKIs on RON, MET expression, cellular signaling and tumor growth in BxPC3 xenograft tumor models. **(A)** Average tumor volumes from nude mice (*n* = 4 per group) inoculated with 5 × 10^6^ BxPC3 cells. ***p < 0.0001. **(B)** A portion of BxPC3 tumor samples from different groups were lysed using tissue lysis buffer as previously described. Proteins (50 μg per sample) were analyzed by western blotting to detect RON, MET, AKT, ERK1/2, phospho-AKT, and phospho-ERK1/2. Phospho-RON and phospho-MET were analyzed by western blotting after immunoprecipitation.

Analysis of the lysate proteins from BxPC3 cell xenograft tumors confirmed that phospho-RON and phospho-MET expression was significantly diminished in the BMS777607-treated groups. The PHA665752-treated groups had significantly diminished phospho-RON expression and slightly diminished phospho-MET expression. Moreover, inhibiting phospho-RON and phospho-MET expression significantly diminished both AKT and ERK1/2 phosphorylation, but had no effect on their protein expression. In the INCB28060-treated groups, inhibiting only phospho-MET expression had no effect on the downstream signaling pathway (Figure 6B).

## Discussion

Pancreatic cancer is a digestive system malignancy of global health concern. Angiogenesis, the presence of highly resistant cancer stem cells, and dysregulation of the cell cycle and apoptosis are thought to be critical in pancreatic cancer chemoresistance (39). Due to the highly proliferative and chemoresistant nature of pancreatic cancer, current therapeutic options such as chemoradiotherapy can do little to improve survival and patient quality of life. At the same time, most patients with pancreatic cancer are diagnosed late and often miss the opportunity for surgery. The development of precision medicine, which can support the personalized therapeutic approach, is likely a future treatment hope for pancreatic cancer. Better understanding of the signaling pathways and mechanisms dysregulated during pancreatic cancer development aid the development of targeted treatment of pancreatic cancer (40).

Studies from the past decade have shown that RON and MET play a role in most known cancer subtypes and are potential markers of poor prognosis and therapeutic target in a number of cancers (41, 42). In the present study, we found increased RON and MET expression in pancreatic cancer tissues, and RON and MET expression were highly correlated. RON and MET were important independent risk factors. As RON and MET expression levels increased, survival became shorter; survival was obviously shorter in RON and MET co-overexpression. Accordingly, RON and MET can be important diagnosis biomarkers and prognosis indicators in pancreatic cancer. However, some studies have shown that RON expression is not associated with prognosis in resected pancreatic cancer (43). There are also some studies showed that elevated MET expression is a strong and independent risk factors of poor patient survival compared to RON, and the MET and RON co-expression does not appear to reflect a synergistic mechanism of reducing pancreatic cancer patient survival times (19). This may be due to the differences in RON expression at RNA and protein level. Moreover, there are many variants of RON, and different antibodies recognize different variants of RON. In addition, IHC staining showed that elevated RON expression in the pancreatic cancer tissue samples was associated with distant metastasis, but that MET expression was associated with tumor size in our study. In a recent study by Babicky ML et al., they found that RON expression can accelerate pancreatic carcinogenesis and loss of functional RON slows progression to pancreatic cancer. They also found RON knockdown significantly inhibits tumor growth *in vivo* (20). In the future, we suggest that the combined detection of RON and MET in tumor tissue has better clinical value for the pathological diagnosis and prognosis evaluation in patients with pancreatic cancer. We assume that RON and MET are suitable targets in pancreatic cancer treatment, and new therapy will further increase survival.

We used four small-molecule TKIs (BMS777607, PHA665752, INCB28060, Tivantinib) to explore the function of RON and MET in pancreatic cancer cells and whether RON and MET could serve as new targets for future treatment of pancreatic cancer. BMS777607 and PHA665752, both targeting RON and MET, could inhibit pancreatic cancer cell viability and migration and induce apoptosis effectively (Figures 2, 3). They also inhibited tumor growth in the pancreatic cancer xenograft tumor model (Figure 5). INCB28060, targeting MET alone, had almost no effect on the cell viability, migration, and tumor growth of the four pancreatic cancer cell lines and the xenograft tumor model. Tivantinib is the first drug to be tested in phase III clinical trials (NCT01755767, METIV-HCC; NCT02029157, JET-HCC) (29). It can affect microtubule dynamics independently of MET, induce G2/M arrest, and induce apoptosis (29). In the present research, we confirm that Tivantinib cannot inhibit the MET and downstream signaling pathways, but that it has a good inhibitory effect on pancreatic cancer cells. *In vivo* and *in vitro* assays all confirmed that TKIs targeting RON had better inhibitory effects and that inhibiting MET alone had almost no effect. These data indicate that survival is obviously shorter in patients with pancreatic cancer with RON and MET co-overexpression, and that TKIs targeting RON and MET, e.g., BMS777607, might be of great significance for treating pancreatic cancer, especially in patients with high RON and MET expression, and for prolonging survival. However, TKIs targeting MET alone had almost no inhibitory effect. RON is more meaningful as a new target of future pancreatic cancer treatment. This may be because RON can mediate oncogenic phenotypes and addiction to KRAS signaling (20). Some researchers have found that “KRAS addiction” is associated with EMT (epithelial to mesenchymal transition) and tumor cell survival (44). Furthermore, RON-mediated EMT is mainly achieved by activating both the RAS–ERK and PI3K–AKT signaling pathways (7). Most of the pancreatic cancer cell lines have KRAS mutations. Therefore, RON, an important KRAS effector, may play a very important role in pancreatic cancer (45). Here, the lack of inhibitory effect by the MET-targeting TKIs was probably related to dosage, drug resistance, and pancreatic cancer cells being highly dependent RON signaling. This report validates the preclinical efficacy of anti-RON and MET for potential targeted therapy of pancreatic cancer.

In addition, many molecular targets have been developed in pancreatic cancer. Erlotinib and gefitinib, TKIs in phase III studies, block EGFR (epidermal growth factor receptor) selectively. Trastuzumab and cetuximab are mAbs against the HER2/neu (erb-b2 receptor tyrosine kinase 2) receptor and EGFR, respectively (46). Moreover, as many targeted agents have been developed, we confirmed that Zt/g4-DM1, as a model of RON-targeted drug delivery for treating pancreatic cancer, is highly effective alone or in combination with chemotherapeutics for inhibiting pancreatic cancer xenograft growth (22).

RON and MET belong to a family of tyrosine kinase receptors and have similar structures. Many researchers have investigated the possible cross-talk between RON and MET (16, 19, 47). There is transphosphorylation between RON and MET and reciprocal regulation of the kinase activity. Homo- and heterodimers of RON and MET are present on the cell surface. Therefore, the formation of a RON and MET complex leads to more efficient RON transphosphorylation by MET, and therefore activates the downstream signaling pathways more effectively (16). There is research revealed BxPC3 cells co-express the RON and MET expression at the cell population level and HGF and MSP can both activate ERK1/2 in BxPC3 cells (19). This is very consistent with our findings. Flow cytometric and western blot analysis showed that the BxPC3 cells had RON and MET co-expression in our study. Here, we also found that RON and MET can be activated by their ligands MSP and HGF, respectively, and further activate the downstream signaling pathways in BxPC3 cells. Next, we found that BMS777607 and PHA665752 inhibition of RON and MET phosphorylation can better inhibit the downstream signaling pathways and that INCB28060 inhibiting MET alone does not inhibit the downstream signaling pathways effectively (Figure 4). These findings confirm the cross-talk between RON and MET. The data also demonstrate that the MSP–RON signaling pathway, but not the HGF–MET signaling pathway, may be the dominant mechanism for cell viability and metastasis.

Moreover, the positive correlation of RON expression with MET has been reported in prostate cancer, and patients with RON and MET co-expression had the lowest 10-years disease-free survival in node-negative breast cancer (48, 49). Others have found that knockdown of MSP-RON signaling delays tumor progression and enhances HGF-MET signaling in pancreatic cancer cell lines (47). HGF–MET and MSP–RON can increase cell migration, but only HGF–MET increases proliferation in BxPC3 cells (19). HGF-MET signaling may play an important role in the invasion and metastasis of pancreatic cancer cells (47). And HGF-MET may be the dominant mechanism mediating EMT in prostate cancer cell lines (48). MET activation can also serve as a primary oncogenic driver or a secondary driver of acquired resistance to targeted therapy in different subsets of lung cancer (42). However, other research has suggested that RON plays a prominent role in both cancer cells and the tumor-associated microenvironment (7). This may be closely related with tumor heterogeneity and complexity. Moreover, RON overexpression can increase gemcitabine resistance in pancreatic cancer, and RON inhibition sensitizes pancreatic ductal adenocarcinoma (PDAC) cells to gemcitabine (20). Many patients with pancreatic cancer with gemcitabine-resistance might benefit from a combination of RON and/or MET inhibitors.

In the present study, we compared the inhibitory effects of four TKIs on human pancreatic cancer cell lines expressing variable levels of RON and MET. We demonstrate clearly that RON and MET can be new therapeutic targets in pancreatic cancer. RON is more meaningful as a new future therapeutic target than MET. However, RON and MET may interact with many other RTKs. In human cancer, the RTKs RON, MET, and EGFR are frequently co-expressed and promote resistance to targeted therapeutics (50). TKIs targeting only RON and MET may not be sufficiently effective. Accordingly, combining the RON-targeting inhibitors with chemotherapy or other targeted agents may achieve a better treatment effect. The present findings uncover the synergism effect between TKIs and gemcitabine via *in vitro* and *in vivo* experiments. In the future, we will evaluate the potential use of these TKIs targeting RON and MET in combination with gemcitabine. Moreover, further understanding of the cross-talk of these receptors may present more possibilities for treating pancreatic cancer.

In conclusion, RON and MET are widely expressed in pancreatic cancer tissues. RON and MET expression are highly correlated with OS in pancreatic cancer. RON and MET may be involved in the malignant process of pancreatic cancer, and can serve as a biomarker for evaluating the prognosis of patients with pancreatic cancer. There is a complex cross-talk between RON and MET. We believe that the MSP–RON signaling pathway, but not the HGF–MET signaling pathway, may be the dominant mechanism in pancreatic cancer. TKIs targeting RON and MET have a better inhibitory effect on pancreatic cancer cell *in vivo* and *vitro* experiment. Increased RON and MET expression by pancreatic cancer cells is a suitable target for anti-RON and anti-MET drugs in future cancer therapy. These findings also provide support for the use of TKIs targeting RON and RON/MET as a novel and potential approach for pancreatic cancer therapy.

## Data Availability Statement

The raw data supporting the conclusions of this manuscript will be made available by the authors, without undue reservation, to any qualified researcher.

## Ethics Statement

The Ethics Committee of The First Affiliated Hospital, Zhejiang University School of Medicine, approved the present study (reference numbers: 2017427-1 and 2017400-1).

## Consent for publication

We have obtained consent to publish this paper from all the participants.

## Author Contributions

Study conceptualization and supervision was carried out by H-PY, X-MX, and M-HW. Sample collection, resources, IHC, and/or clinical characterization were conducted by H-PY, C-YH, X-MX, BH, Z-GW, YQ, and T-HW. The *in vitro* cellular experiments and data analysis were performed by C-YH, T-HW, T-MT, and Y-ZL. The animal experiments were carried out by C-YH, T-MT, and Y-ZL. The manuscript was drafted by H-PY, M-HW, and C-YH. All authors read and approved the manuscript.

### Conflict of Interest

The authors declare that the research was conducted in the absence of any commercial or financial relationships that could be construed as a potential conflict of interest.

## Abbreviations

ADC Zt/g4-DM1: Monoclonal antibody (mAb) Zt/g4-drug maytansinoid conjugates
ATCC: American type culture collection
CI: Confidence interval
ELISA: Enzyme-linked immunosorbent assay
EMT: Epithelial to mesenchymal transition
ERK: Extracellular signal-regulated kinases
EGFR: Epidermal growth factor receptor
FBS: Fetal bovine serum
FITC: Fluorescein isothiocyanate
FTI: Farnesyltransferase inhibitor
HGF: Hepatocyte growth factor
IC50: Half maximal inhibitory concentration
IHC: Immunohistochemical
MAP: Mitogen-activated protein
MET: Hepatocyte growth factor receptor
MFI: Median fluorescence level
MST1R: Macrophage stimulating 1 receptor
mAb: Monoclonal antibody
MSP: Macrophage-stimulating protein
OS: Overall survival
PBS: Phosphate-buffered saline
PI-3K: Phosphatidylinositol 3-kinases
RON: Recepteur d'origine nantais
RTK: Receptor tyrosine kinase
RR: Relative risk
RLU: Relative light unit
TKI: Tyrosine kinase inhibitors
VEGF: Vascular endothelial growth factor.
