# Supplementary material for: Aberrant RON and MET Co-overexpression as Novel Prognostic Biomarkers of Shortened Patient Survival and Therapeutic Targets of Tyrosine Kinase Inhibitors in Pancreatic Cancer
Source: Front Oncol. 2019 Dec 5;9:1377. doi: 10.3389/fonc.2019.01377 (PMC6906148; doi:10.3389/fonc.2019.01377)
Supplement: Supplementary file 1 [file Data_Sheet_1.ZIP › supplementary file/supplementary file1.pdf]

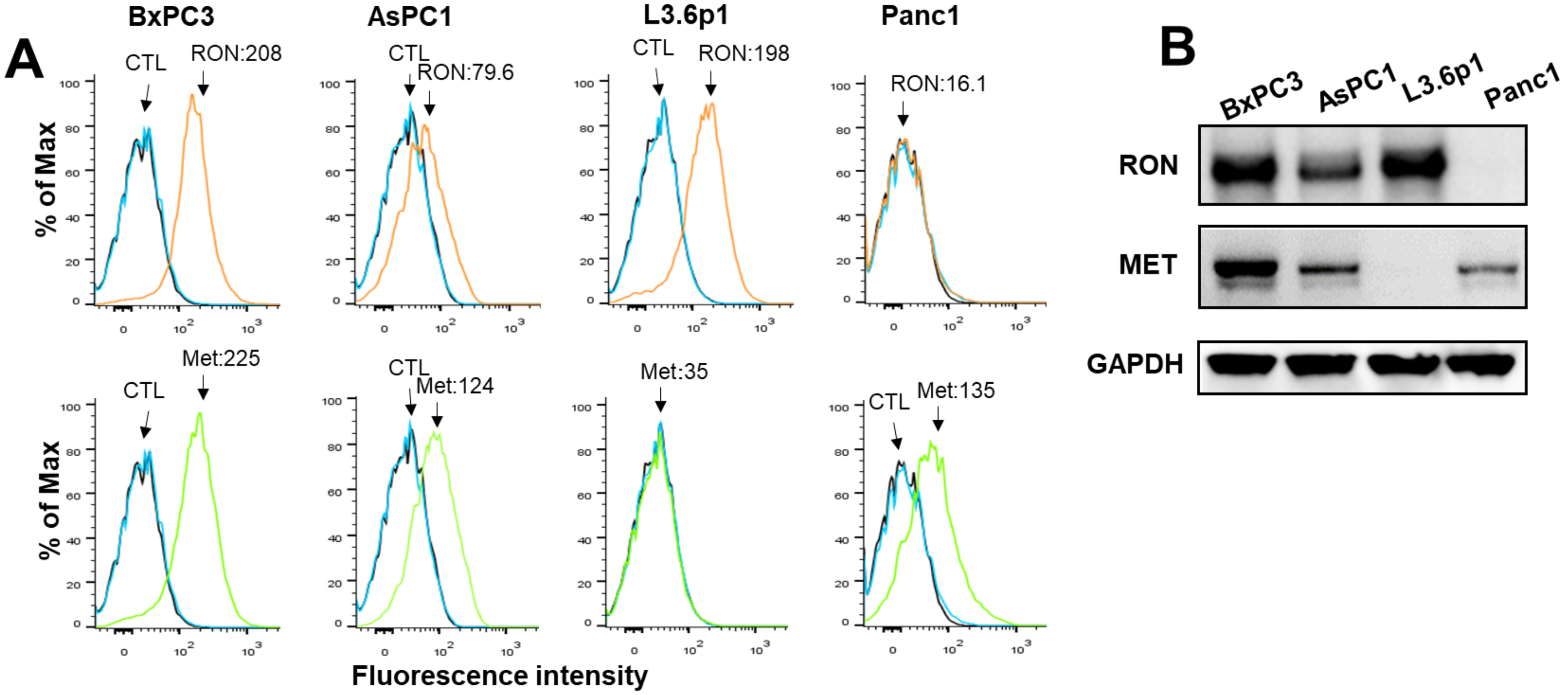

Supplementary file 1

Figure S1. (A) Flow cytometric identification of pancreatic cancer cell lines expressing RON and MET levels. Four pancreatic cancer cell lines (1×10^6 cells/mL) in PBS were incubated at 37°C with 5 µg/mL anti-RON mAb (Zt/g4) or anti-MET mAb (ab51067; 1:100) for 60 min, and labeled with fluorescein isothiocyanate (FITC). Isotype-matched mouse IgG was used as the control. Fluorescence intensity of individual samples was determined by flow cytometric analysis. (B) Western blot of BxPC3, AsPC1, L3.6p1, Panc1 cells. Proteins analyzed include RON and MET. The membranes were also reprobed for GAPDH as the loading control.
