# Supplementary material for: Aberrant RON and MET Co-overexpression as Novel Prognostic Biomarkers of Shortened Patient Survival and Therapeutic Targets of Tyrosine Kinase Inhibitors in Pancreatic Cancer
Source: Front Oncol. 2019 Dec 5;9:1377. doi: 10.3389/fonc.2019.01377 (PMC6906148; doi:10.3389/fonc.2019.01377)
Supplement: Supplementary file 1 [file Data_Sheet_1.ZIP › supplementary file/supplementary file2.pdf]

Table S1 Multivariate analysis of overall survival

Variable	RR	95% CI	P-value
**Age**	1.407	1.060-1.868	0.018
**Gender**	1.291	0.958-1.741	0.094
**Tumor size**	1.245	1.069-1.451	0.005
**Lymph node metastasis**	1.113	0.816-1.516	0.499
**Distant metastasis**	2.910	1.882-4.498	0.000
**TNM stage**	0.966	0.781-1.194	0.747
**Differentiation**	0.910	0.705-1.175	0.472
**Chemotherapy**	0.602	0.455-0.795	0.000
**RON**	1.911	1.271-2.875	0.002

Variable	RR	95% CI	P-value
**Age**	1.399	1.055-1.854	0.020
**Gender**	1.225	0.910-1.649	0.180
**Tumor size**	1.284	1.102-1.496	0.001
**Lymph node metastasis**	1.059	0.776-1.443	0.719
**Distant metastasis**	3.015	1.950-4.661	0.000
**TNM stage**	0.939	0.759-1.161	0.560
**Differentiation**	0.908	0.702-1.174	0.460
**Chemotherapy**	0.618	0.468-0.817	0.001
**MET**	1.967	1.193-3.244	0.008

Variable	RR	95% CI	P-value
**Age**	1.359	1.023-1.805	0.034
**Gender**	1.257	0.933-1.693	0.133
**Tumor size**	1.247	1.070-1.453	0.005
**Lymph node metastasis**	1.095	0.802-1.495	0.569
**Distant metastasis**	2.889	1.867-4.472	0.000
**TNM stage**	0.964	0.779-1.193	0.735
**Differentiation**	0.931	0.721-1.202	0.584
**Chemotherapy**	0.608	0.460-0.803	0.000
**RON-MET**	1.664	1.169-2.369	0.005

Supplementary file 2
