# Supplementary material for: Aberrant RON and MET Co-overexpression as Novel Prognostic Biomarkers of Shortened Patient Survival and Therapeutic Targets of Tyrosine Kinase Inhibitors in Pancreatic Cancer
Source: Front Oncol. 2019 Dec 5;9:1377. doi: 10.3389/fonc.2019.01377 (PMC6906148; doi:10.3389/fonc.2019.01377)
Supplement: Supplementary file 1 [file Data_Sheet_1.ZIP › supplementary file/supplementary file3.pdf]

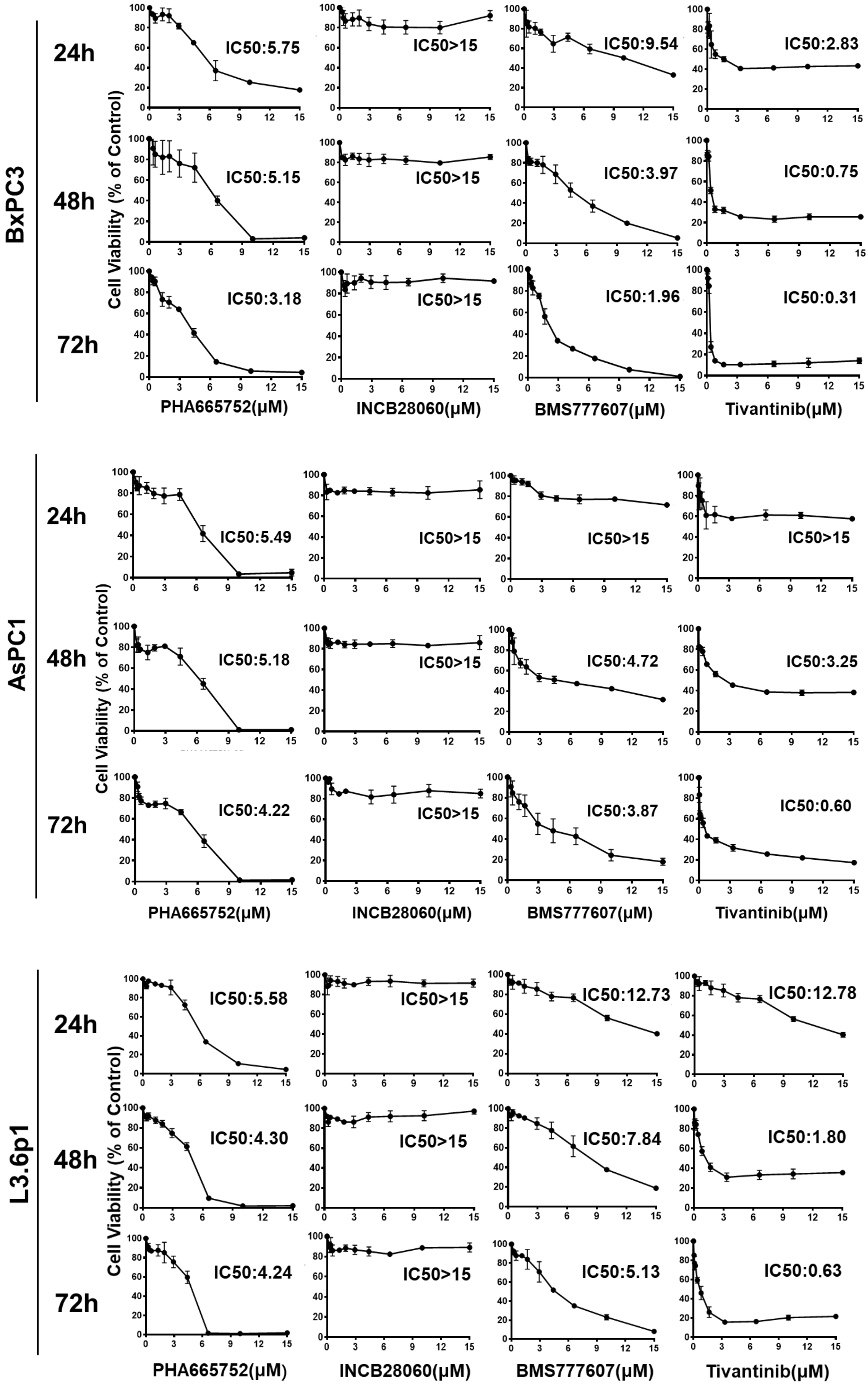

BxPC3
24h
48h
72h
Cell Viability (% of Control)
IC50:5.75
IC50>15
IC50:9.54
IC50:2.83
IC50:5.15
IC50>15
IC50:3.97
IC50:0.75
IC50:3.18
IC50>15
IC50:1.96
IC50:0.31
PHA665752(μM)
INCB28060(μM)
BMS777607(μM)
Tivantinib(μM)
AsPC1
24h
48h
72h
Cell Viability (% of Control)
IC50:5.49
IC50>15
IC50>15
IC50>15
IC50:5.18
IC50>15
IC50:4.72
IC50:3.25
IC50:4.22
IC50>15
IC50:3.87
IC50:0.60
PHA665752(μM)
INCB28060(μM)
BMS777607(μM)
Tivantinib(μM)
L3.6p1
24h
48h
72h
Cell Viability (% of Control)
IC50:5.58
IC50>15
IC50:12.73
IC50:12.78
IC50:4.30
IC50>15
IC50:7.84
IC50:1.80
IC50:4.24
IC50>15
IC50:5.13
IC50:0.63
PHA665752(μM)
INCB28060(μM)
BMS777607(μM)
Tivantinib(μM)

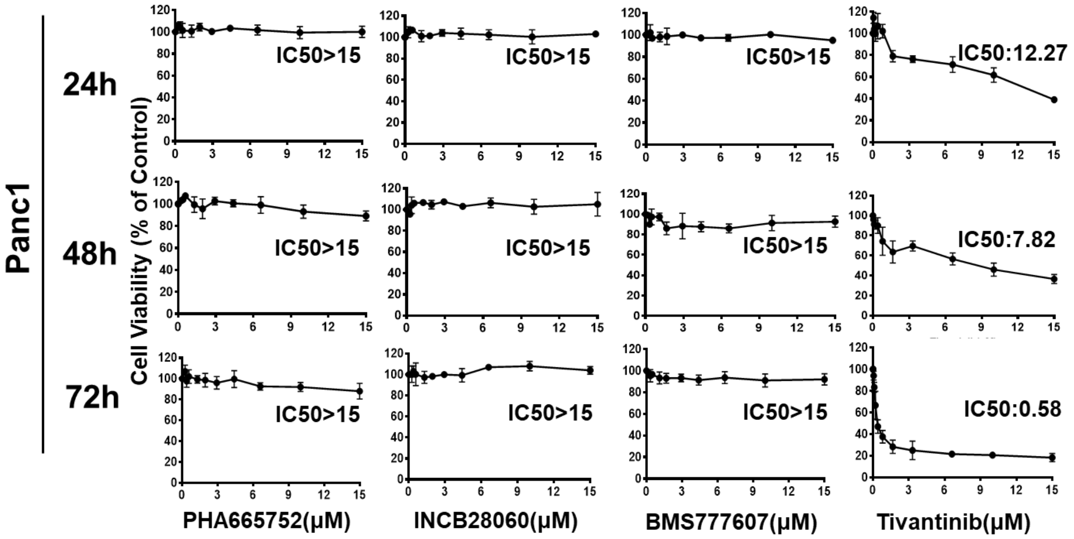

Supplementary file 3

Figure S2. Effect of TKIs on pancreatic cancer cell viability. CCK-8 measurement of pancreatic cancer cell viability following 24-, 48-, and 72-h TKI treatment. Viability of control cells was defined as 100% and used to calculate the percentages of cell viability of the treated cells.
