# Supplementary material for: Aberrant RON and MET Co-overexpression as Novel Prognostic Biomarkers of Shortened Patient Survival and Therapeutic Targets of Tyrosine Kinase Inhibitors in Pancreatic Cancer
Source: Front Oncol. 2019 Dec 5;9:1377. doi: 10.3389/fonc.2019.01377 (PMC6906148; doi:10.3389/fonc.2019.01377)
Supplement: Supplementary file 1 [file Data_Sheet_1.ZIP › supplementary file/Supplementary file4.pdf]

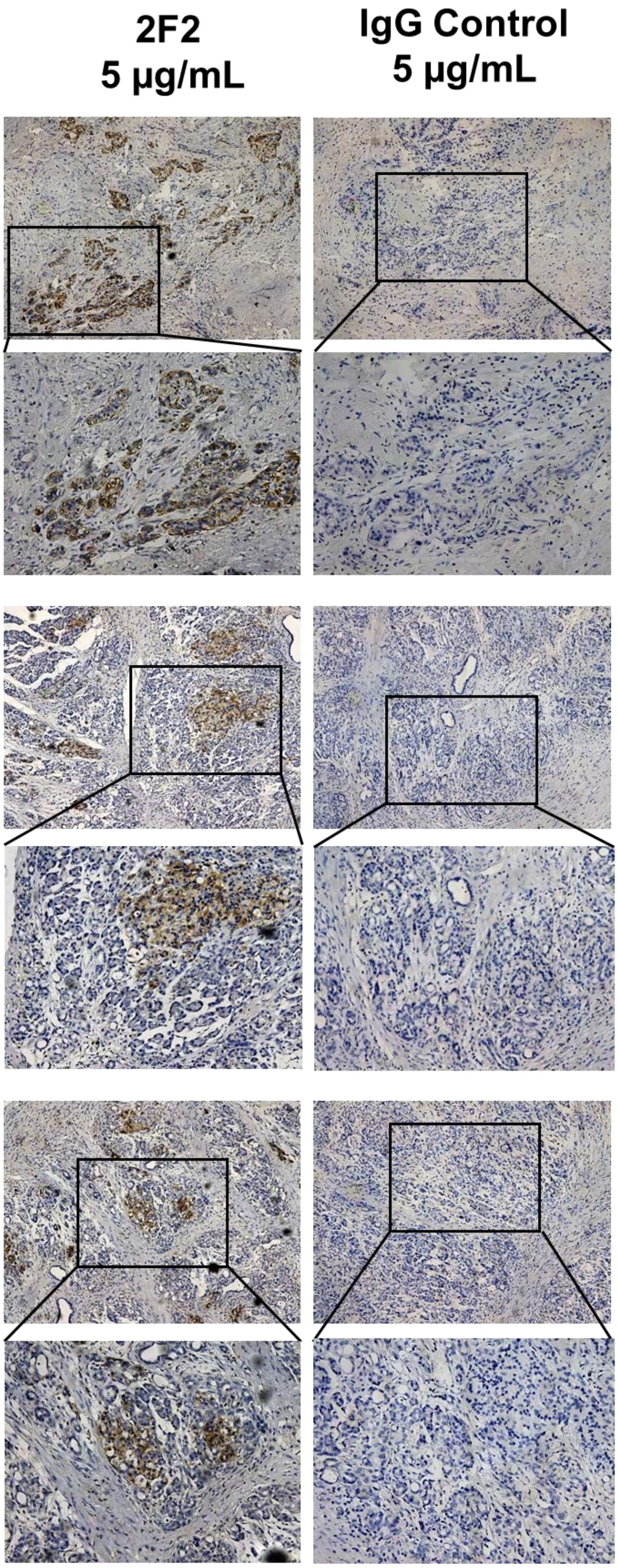


**Supplementary file 4**

Figure S3: IHC staining was carried out using Zt/f2 (5 μg/mL) as the primary antibody for RON**.** Isotype-matched mouse IgG (5 μg/mL) was used as the control. Original magnification ×100 (all photomicrographs).
