# Supplementary figures and images for: Aberrant RON and MET Co-overexpression as Novel Prognostic Biomarkers of Shortened Patient Survival and Therapeutic Targets of Tyrosine Kinase Inhibitors in Pancreatic Cancer
Source: Front Oncol. 2019 Dec 5;9:1377. doi: 10.3389/fonc.2019.01377 (PMC6906148; doi:10.3389/fonc.2019.01377)

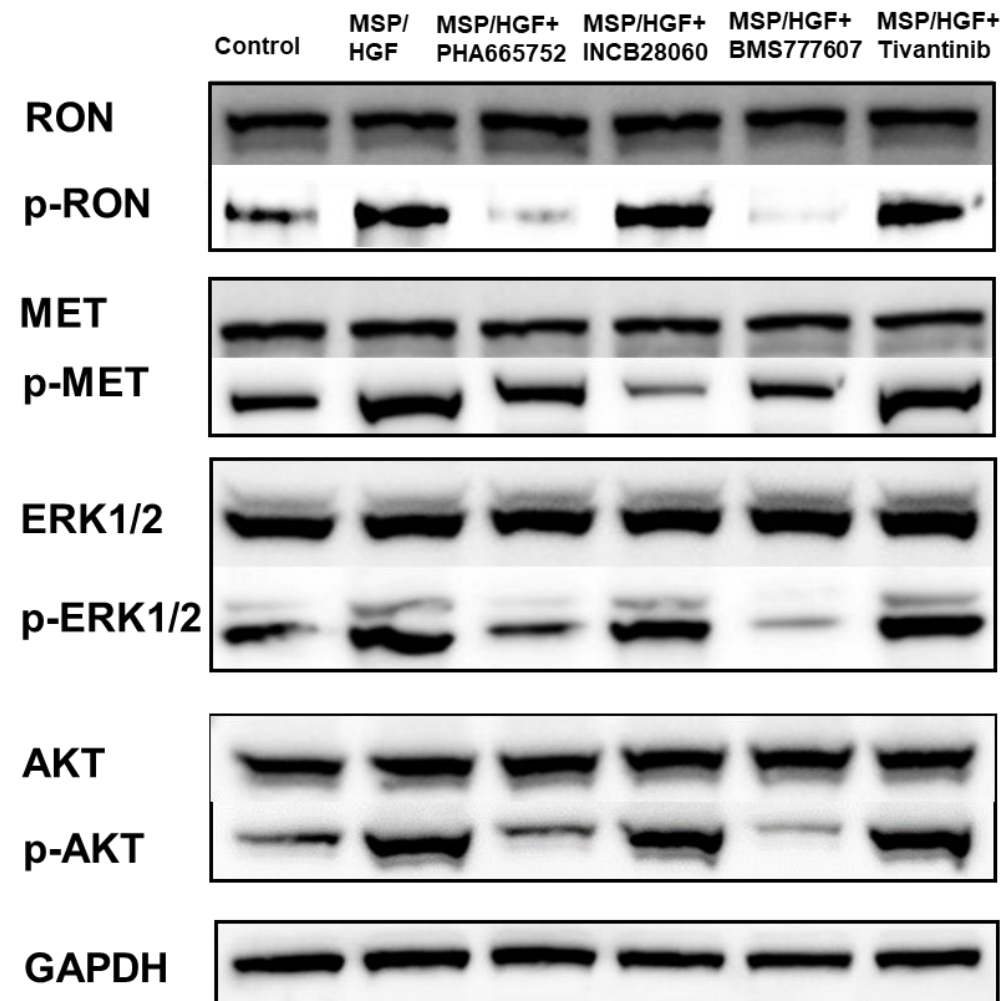

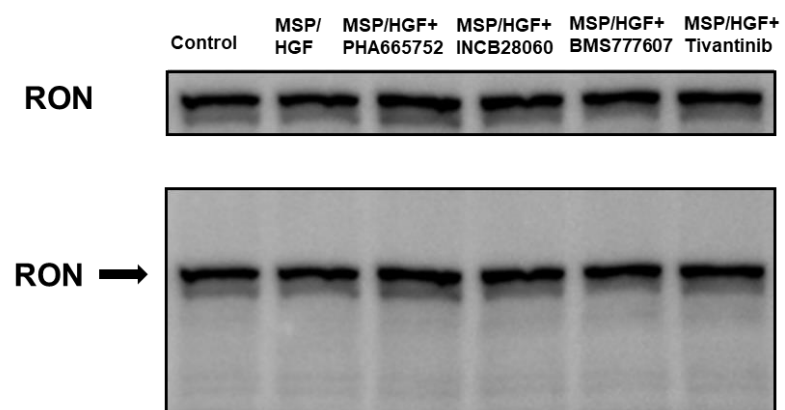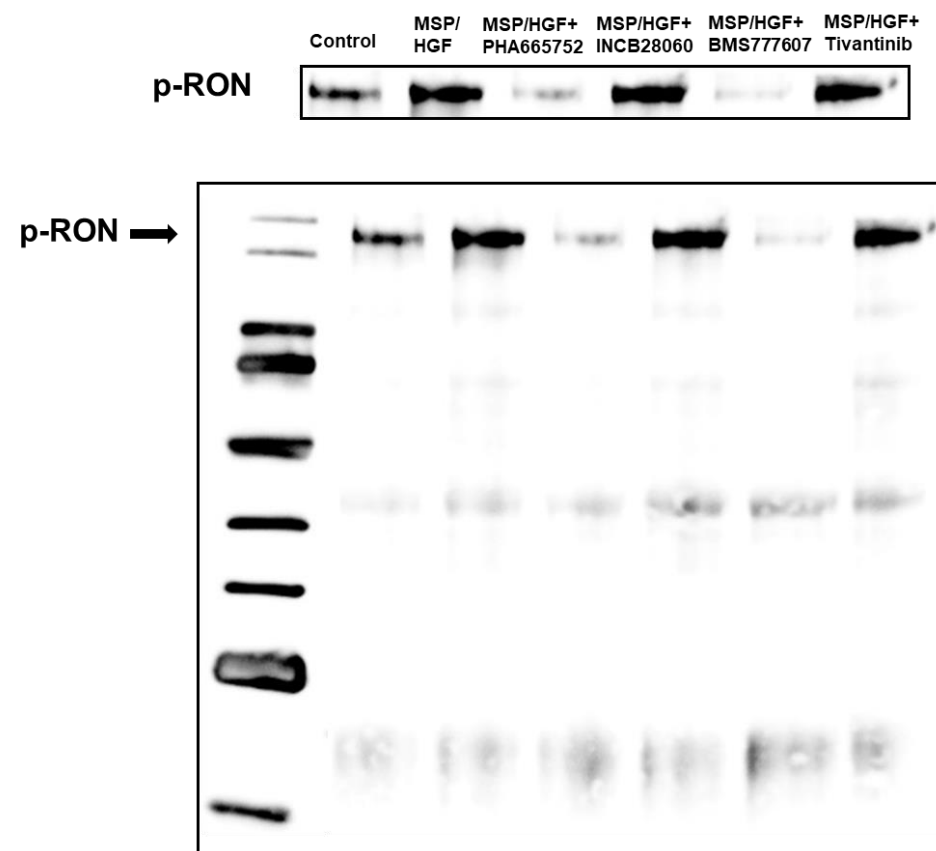

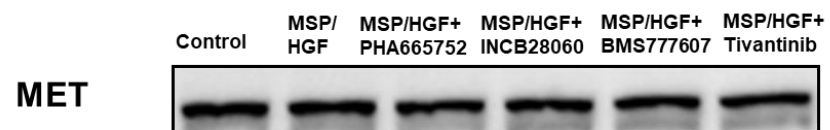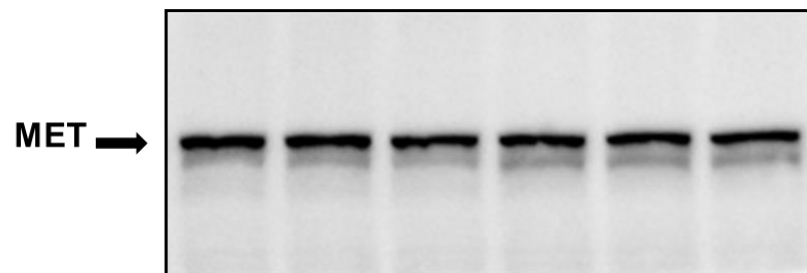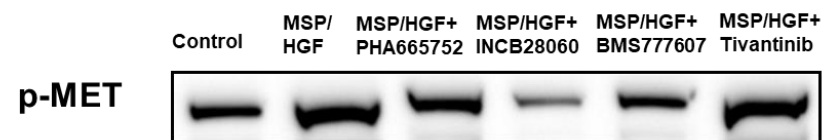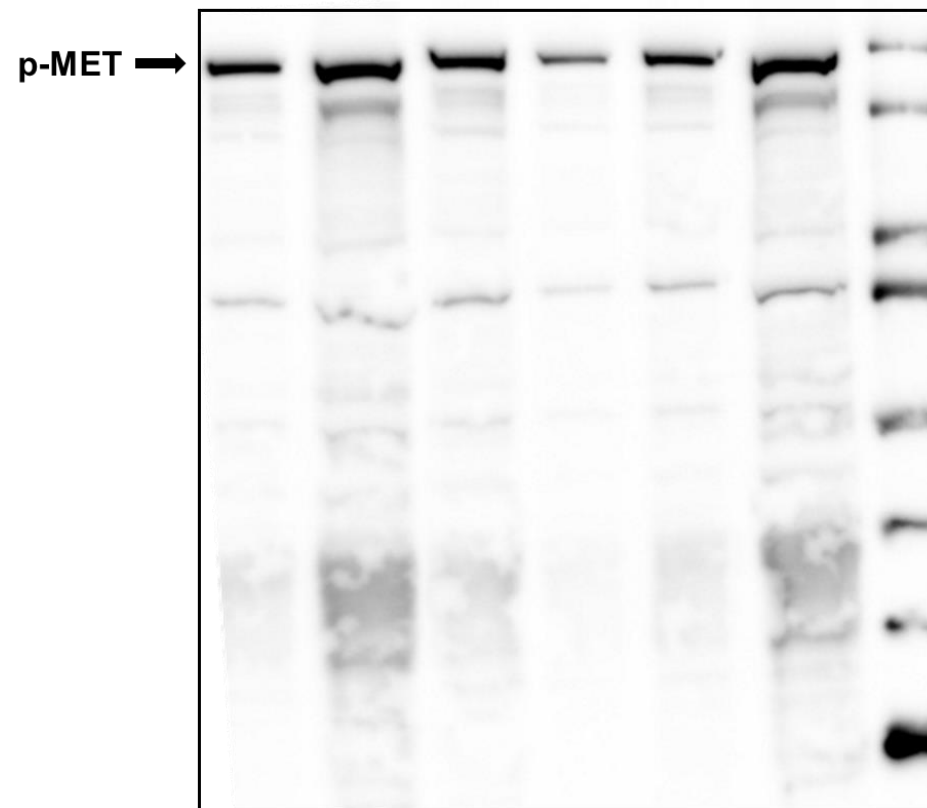

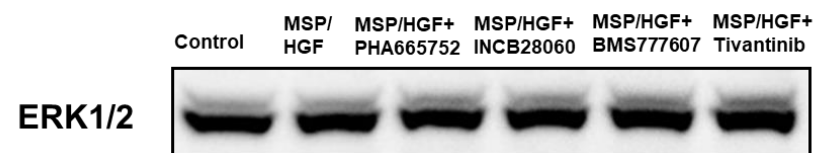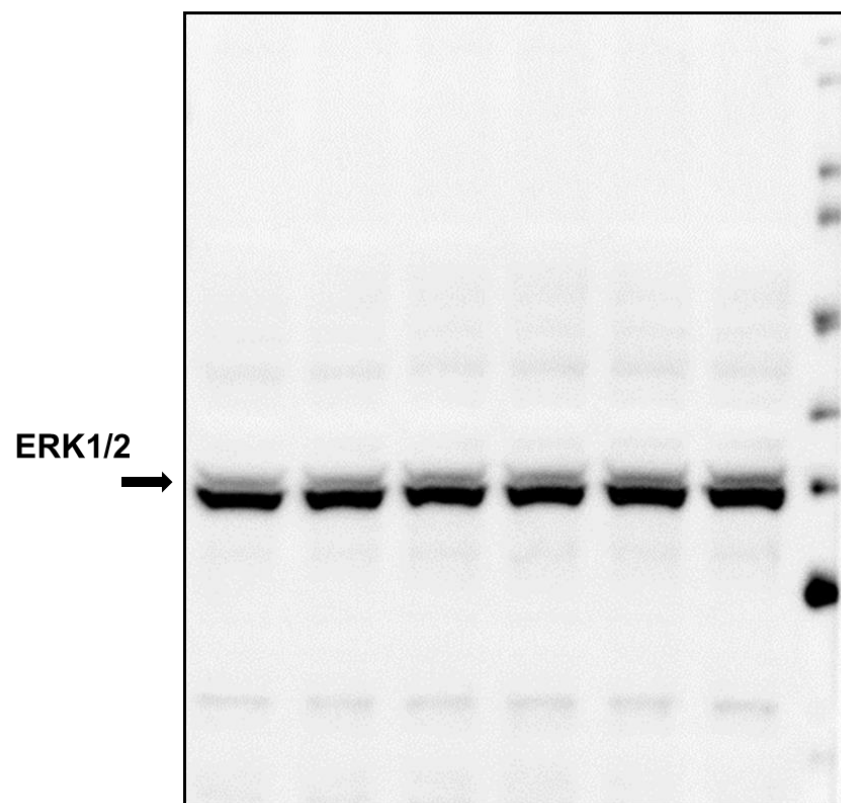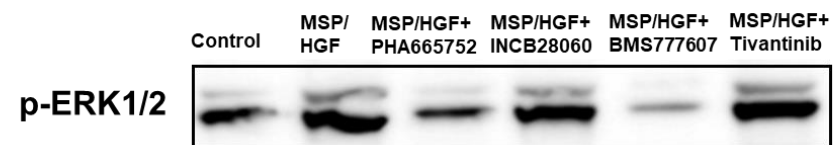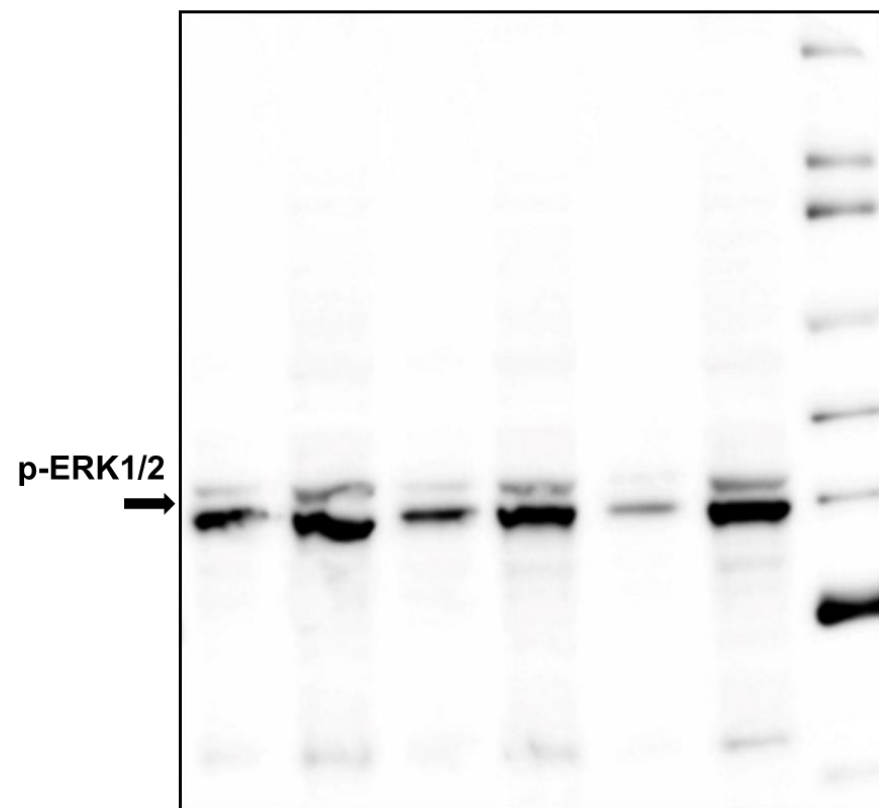

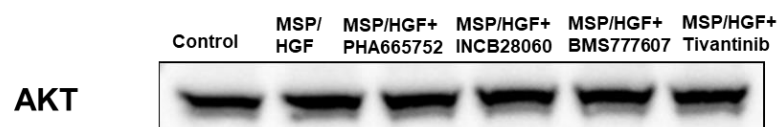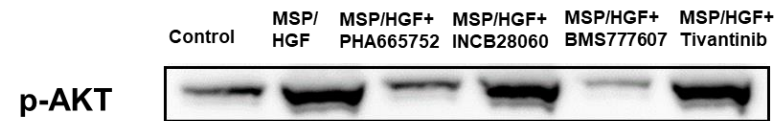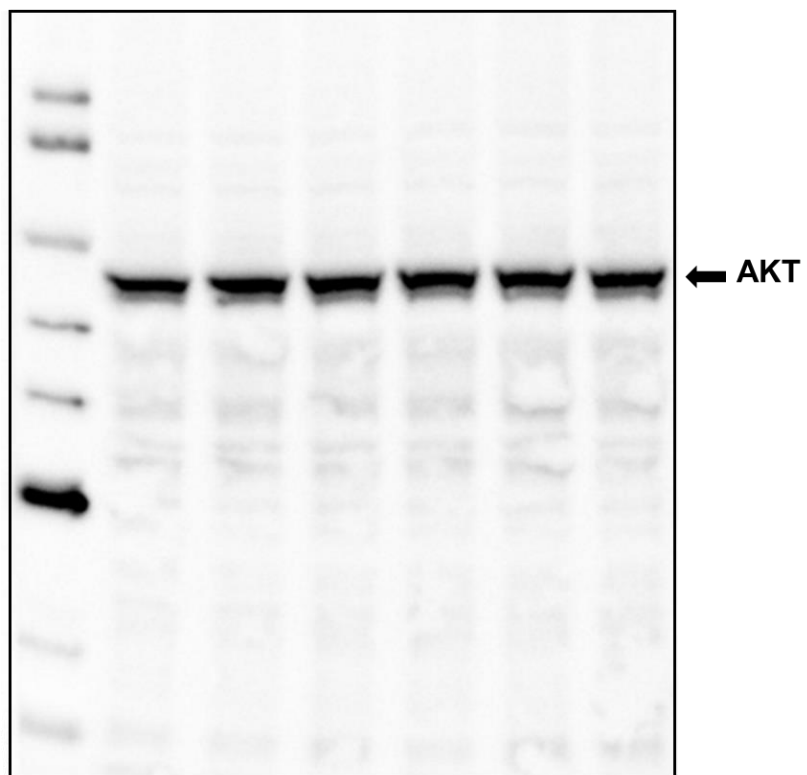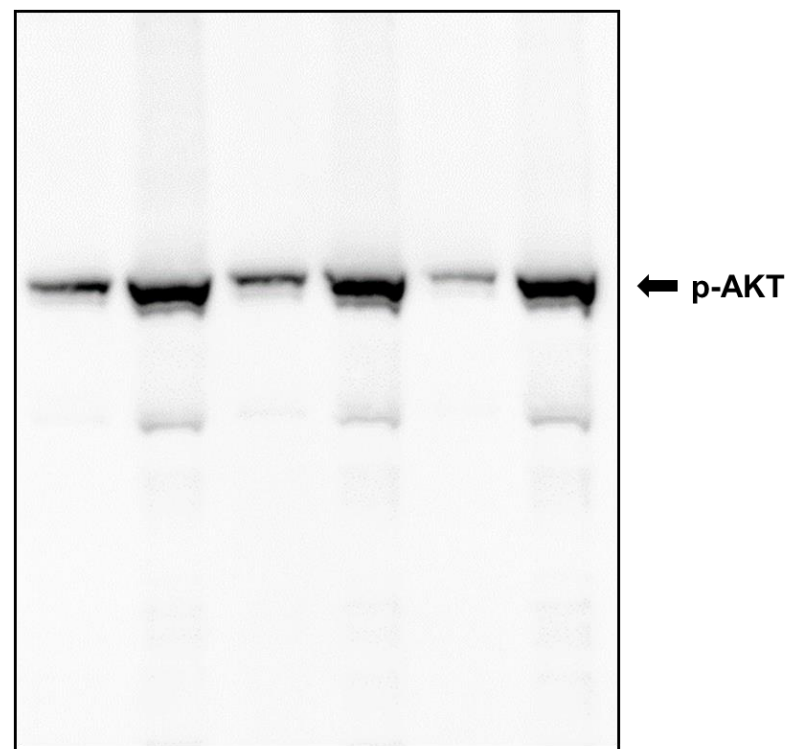

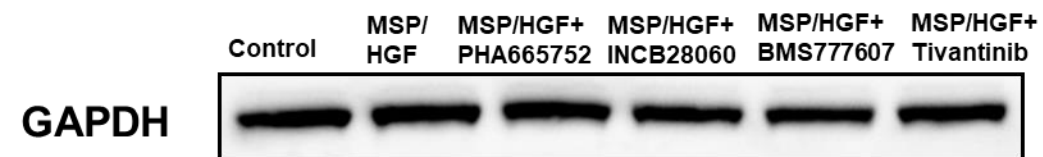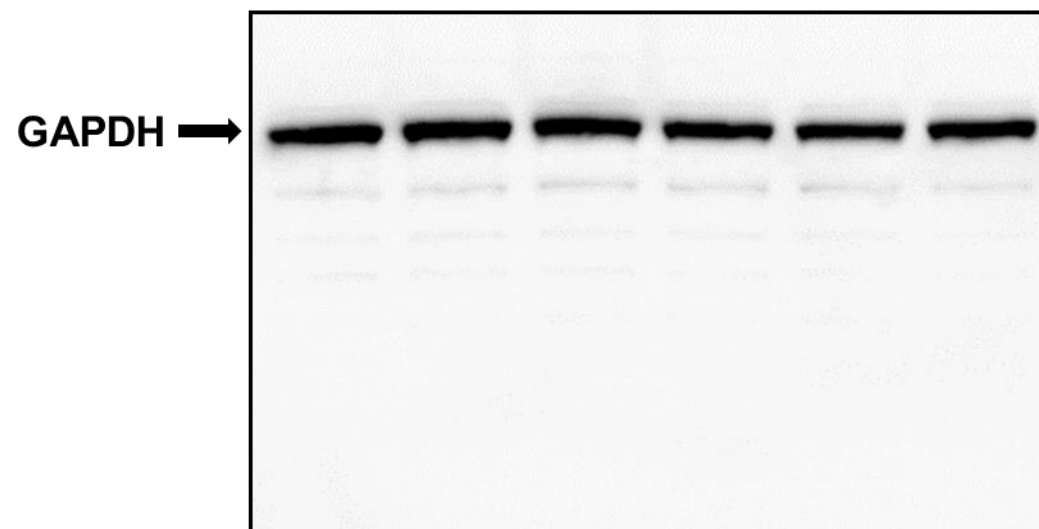

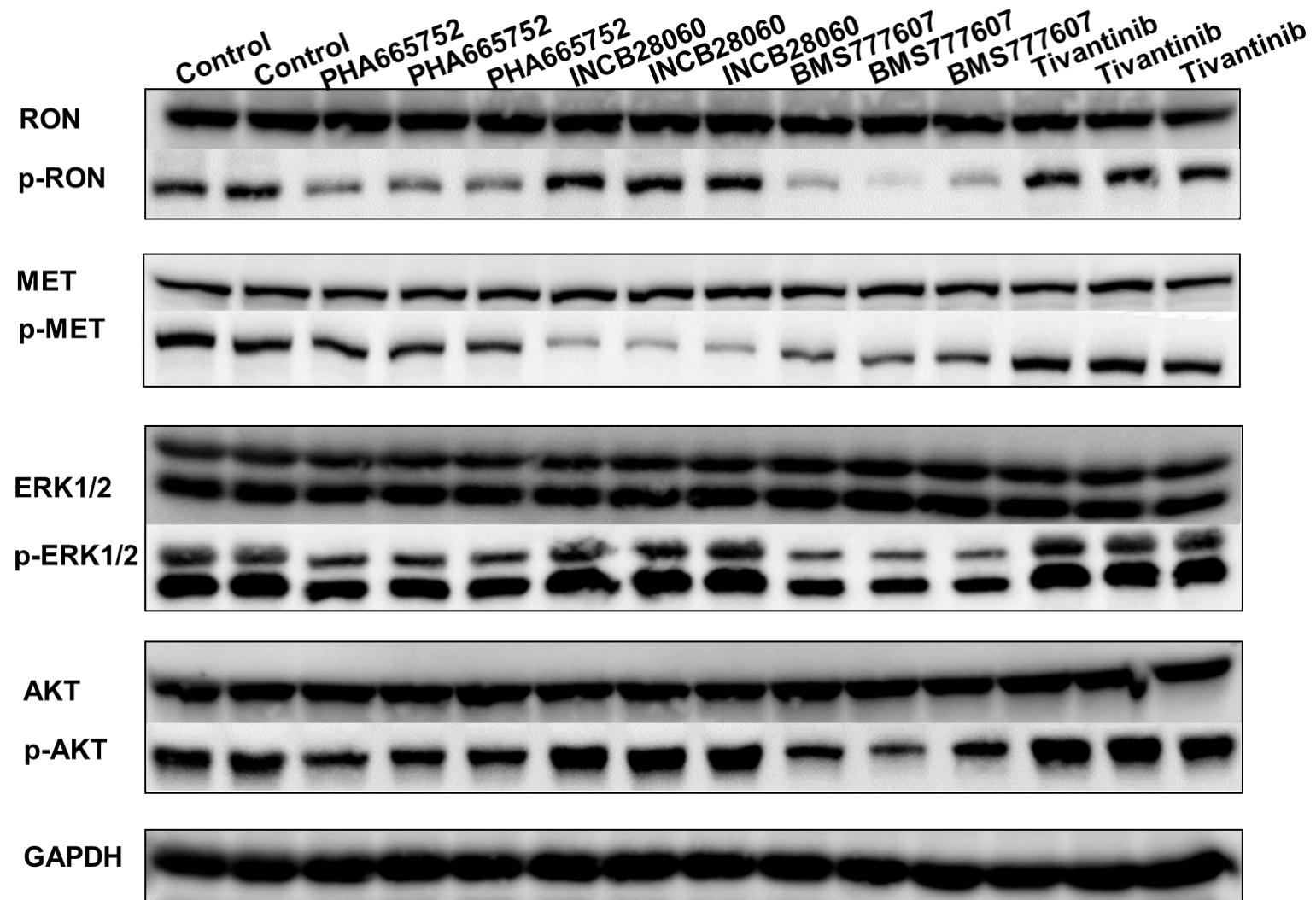

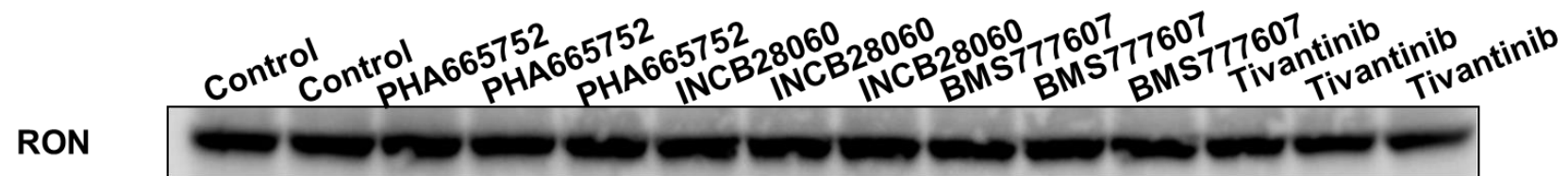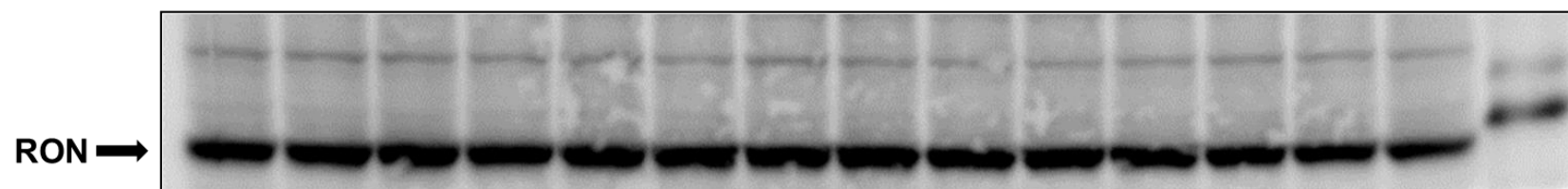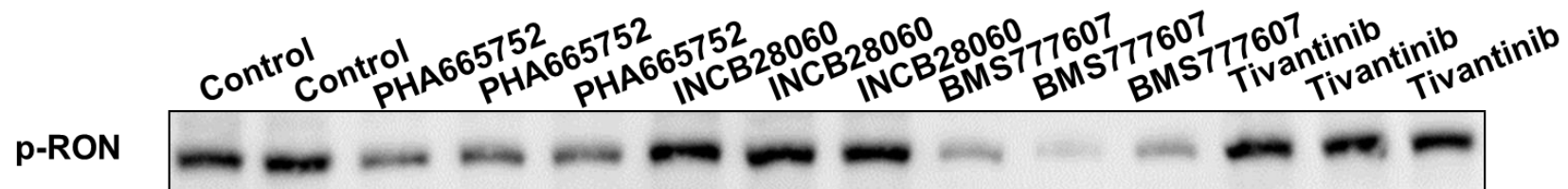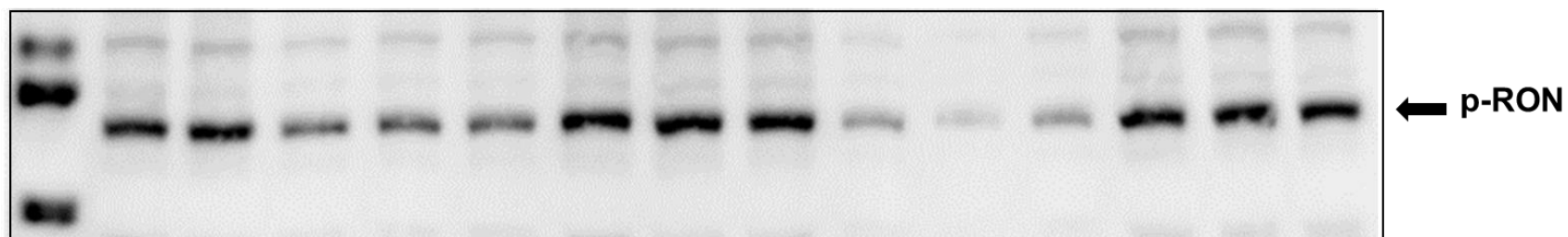

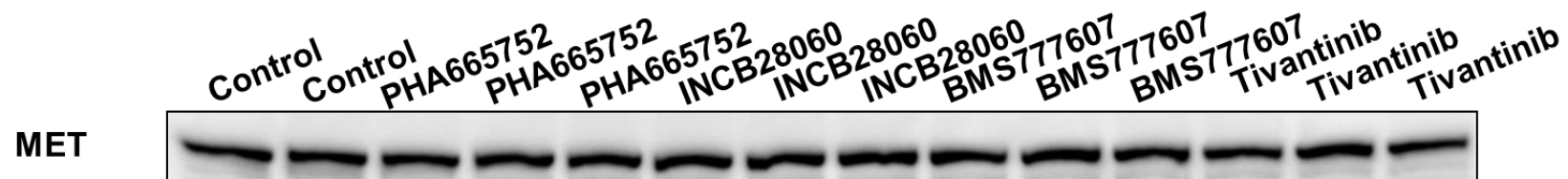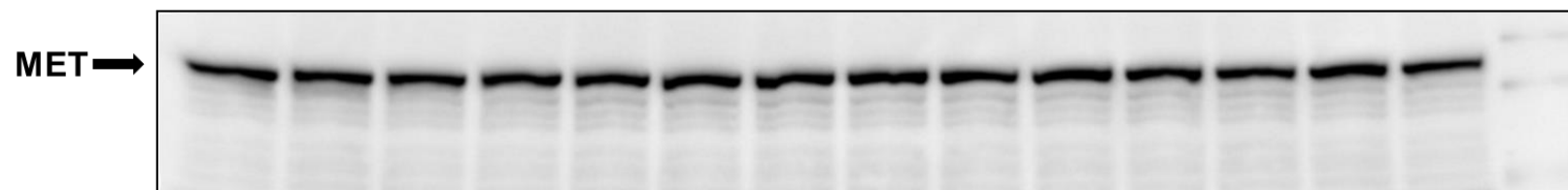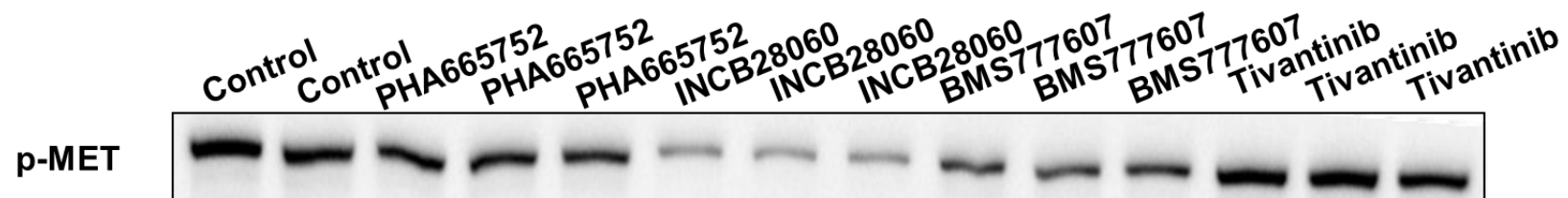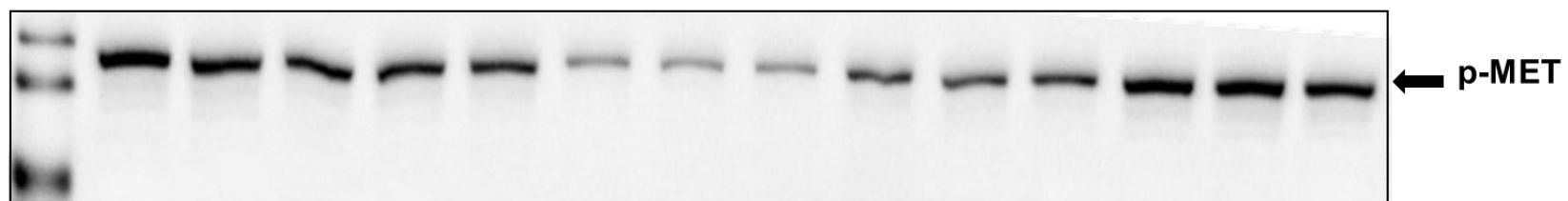

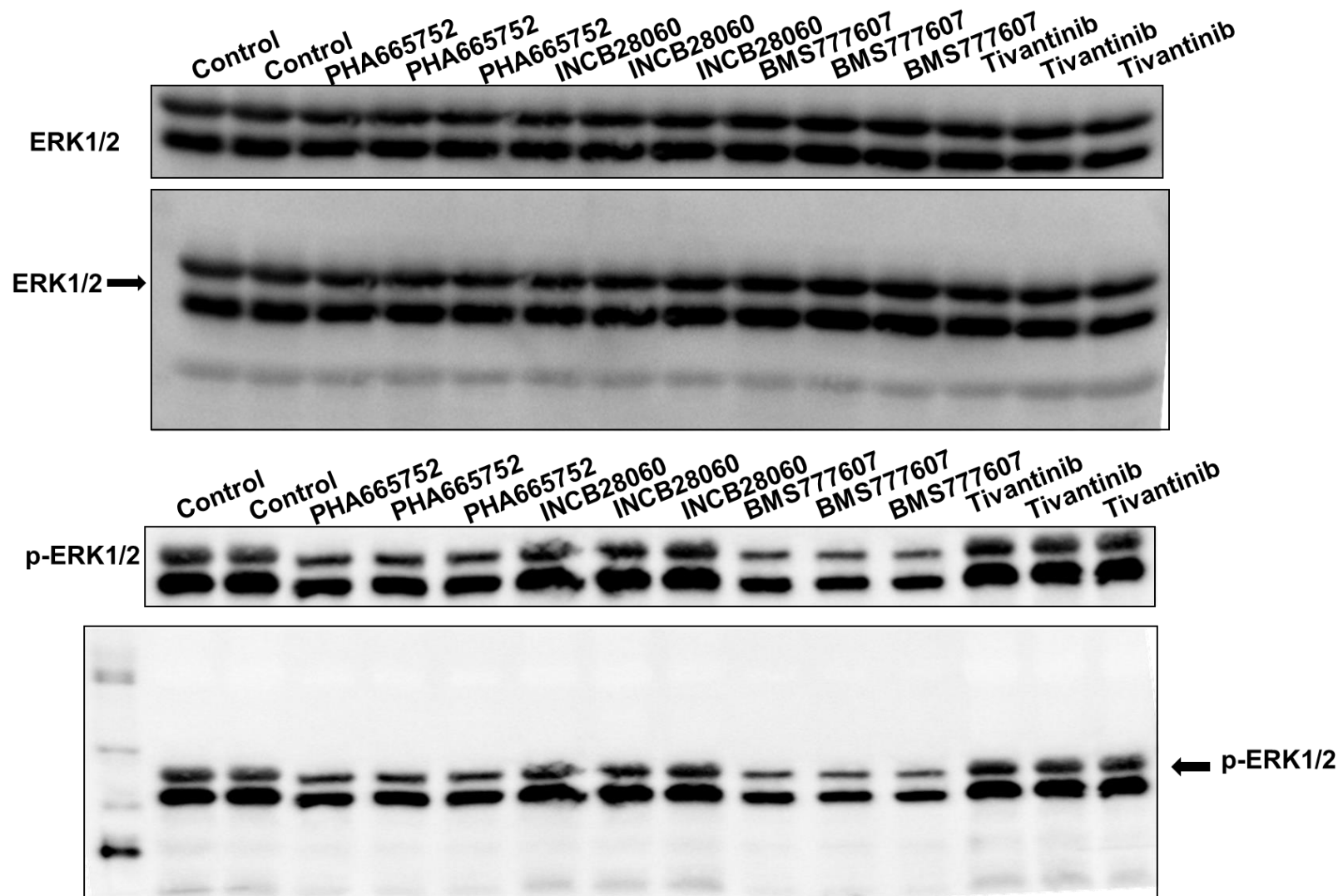

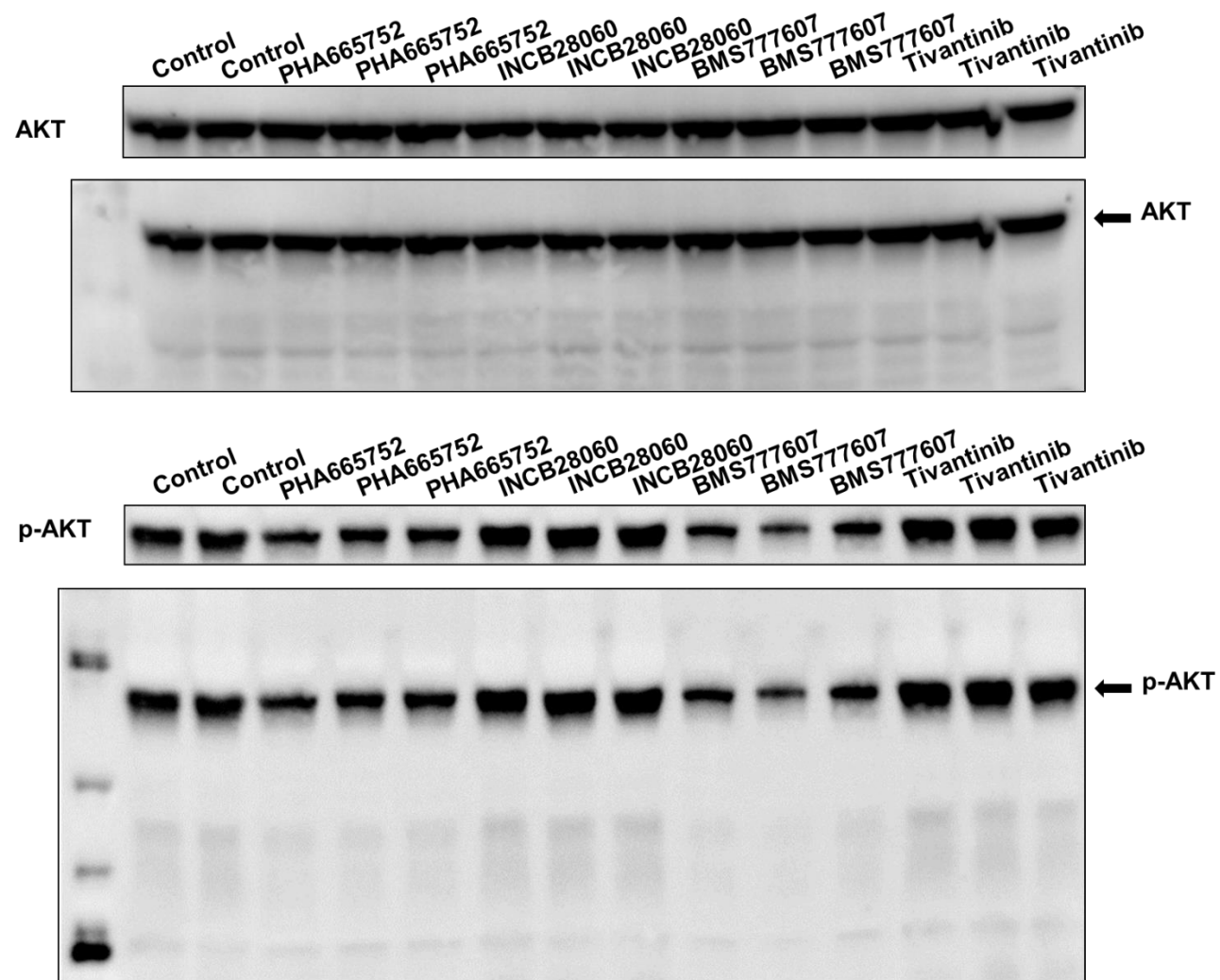

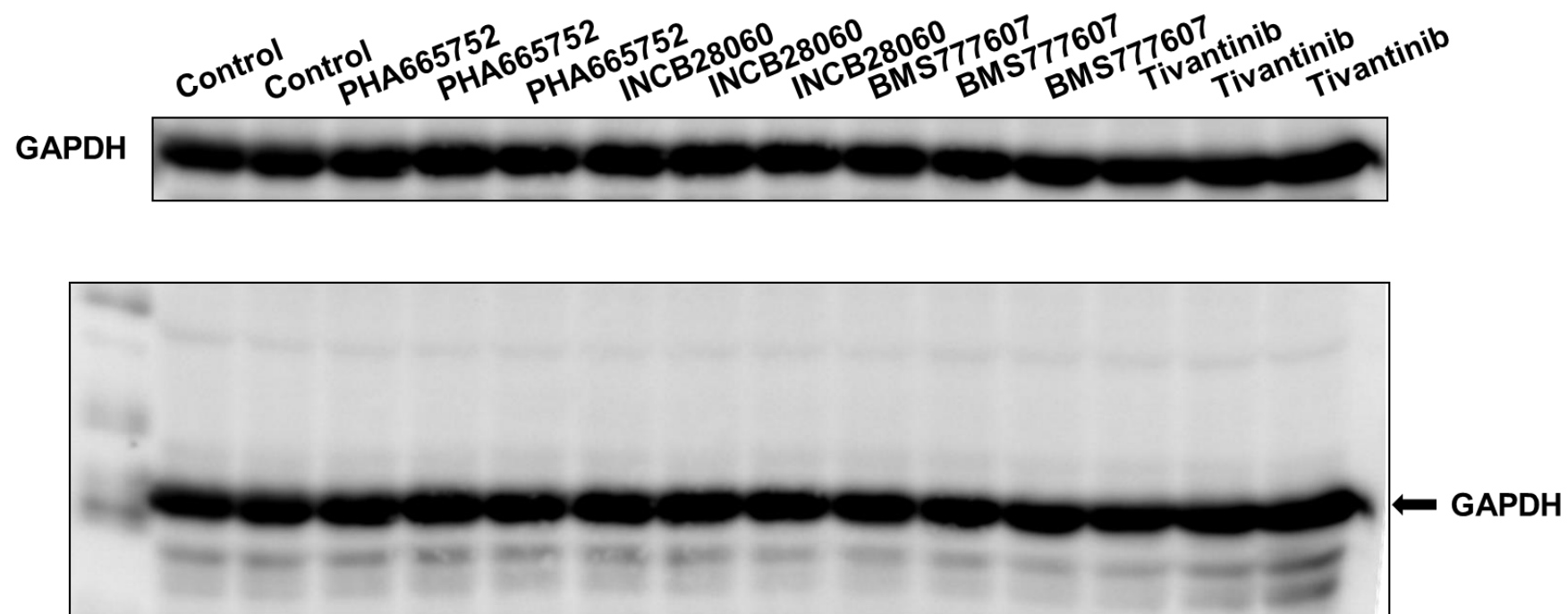

Supplement: Supplementary file 1 [file Data_Sheet_1.ZIP › supplementary file/supplementary file5 western blot original figure.pdf]
